# Supplementary material for: Intervention treatment distributions that depend on the observed treatment process and model double robustness in causal survival analysis
Source: Stat Methods Med Res. 2023 Jan 4;32(3):509–23. doi: 10.1177/09622802221146311 (PMC9983057; doi:10.1177/09622802221146311)
Supplement: sj-pdf-1-smm-10.1177_09622802221146311 - Supplemental material for Intervention treatment distributions that depend on the observed treatment process and model double robustness in causal survival analysis [file sj-pdf-1-smm-10.1177_09622802221146311.pdf]

WEB APPENDIX FOR "INTERVENTION DISTRIBUTIONS THAT DEPEND ON THE OBSERVED TREATMENT PROCESS AND MODEL DOUBLE ROBUSTNESS IN CAUSAL SURVIVAL ANALYSIS"

A. Examples of intervention treatment distributions that depend on the observed treatment process

In this section, we review examples of intervention distributions considered in the literature that have depended on the observed treatment process:

- *Dynamic treatment initiation strategies with grace period:*

  Motivated by questions about the effects of CD4-based treatment initiation strategies, previous authors have considered strategies of the form "If a condition for treatment initiation is met by interval j then start treatment by $j+m$ for a selected grace period m, with no intervention in intervals j through $j+m-1$. Otherwise, do not start at j", $\forall j$ Cain et al. (2010); Young et al. (2011). Thus, for A_j an indicator of treatment initiation by j and $L^*_j \in \mathbf{L}_j$ a monotonic indicator that the condition for initiating treatment has been met by j, under this intervention $A^{g+}_j=1$ when $L^{*g}_{j-m}=1$, $A^{g+}_j=0$ when $L^{*g}_j=0$ and A^{g+}_j equals the natural treatment at time j otherwise. Moreover, it can be shown that the intervention treatment distribution at each j is

  $$q^g(1|1,\bar{\boldsymbol{l}}_j,\bar{a}_{j-1})=\begin{cases}1, & \text{if } l^*_{j-m}=1\\ 0, & \text{if } l^*_j=0\\ f(1|1,\bar{\boldsymbol{l}}_j,\bar{a}_{j-1}), & \text{otherwise}\end{cases}$$

  or $q^g(a_j|1,\bar{\boldsymbol{l}}_j,\bar{a}_{j-1})=(1-l^*_j)(1-a_j)+l^*_{j-m}a_j+(1-l^*_{j-m})l^*_j f(a_j|1,\bar{\boldsymbol{l}}_j,\bar{a}_{j-1})$.

- *Representative interventions:*

  Motivated by observational studies to understand the long-term effects of lifestyle interventions (e.g., interventions that increase daily minutes of physical activity), previous authors have considered *representative interventions* that assign the value of a multi-level treatment to an individual at each $j=0,\ldots,J$ as a random draw from a particular distribution. Specifically, the observed distribution of treatment in interval j among those who, in the observational study, (i) had the same measured confounder and treatment history prior to j as that individual and (ii) had treatment at j at or above a cutoff δ (or more generally, within a pre-specified range), e.g., "at least 30 minutes of daily physical activity" Picciotto et al. (2012); Young et al. (2019). In this case,

  $$q^g(a_j|1,\bar{\boldsymbol{l}}_j,\bar{a}_{j-1})=f(a_j|1,\bar{\boldsymbol{l}}_j,\bar{a}_{j-1},A_j\geq\delta).$$

  This intervention distribution notably only depends on the observed treatment process at j *among those with treatment in the pre-specified range at j*.

- *Deterministic interventions that depend on the natural value of treatment:*

  Alternative interventions that maintain a multi-level treatment within a pre-specified range have been posed that assign treatment at each j as a function of the natural treatment value at j Robins et al. (2004); Taubman et al. (2009), e.g., "If the natural value of treatment at j is below δ then intervene and set treatment at j to δ. Otherwise, do not intervene at j". The resulting intervention distribution at each j (conditional only on the measured past and marginal with respect to the

natural value of treatment at j) is

$$q^g(a_j|1,\bar{\boldsymbol{l}}_j,\bar{a}_{j-1})=F_{A_j}(\delta|1,\bar{\boldsymbol{l}}_j,\bar{a}_{j-1})I(a_j=\delta)+I(a_j\geq\delta)f(a_j|1,\bar{\boldsymbol{l}}_j,\bar{a}_{j-1})$$

where $F_{A_j}(\delta|1,\bar{\boldsymbol{l}}_j,\bar{a}_{j-1})=\sum_{a_j<\delta}f(a_j|1,\bar{\boldsymbol{l}}_j,\bar{a}_{j-1})$.[1] For more detailed calculations and related examples, see Appendix B and Rotnitzky (2013), Young et al. (2014) and Diaz et al. (2021) Haneuse and Rotnitzky (2013); Young et al. (2014); Díaz et al. (2020).

[1]Recall that the intervention treatment distribution as generally defined in Section 3, is the distribution of treatment under intervention conditional *only on the measured past* with "measured past" referring to the past covariates $\overline{\mathbf{L}}_k$. This example is distinct from the previous examples in that the rule for assigning treatment under intervention depends on *more* than these covariates – it also depends on an individual's natural values of treatment. In the previous examples, the intervention treatment distribution can be obtained directly from the statement of the treatment rule because the intervention rule depends on no more than $\overline{\mathbf{L}}_k$. However, in this example, the treatment rule depends on the natural treatment value. Therefore, to calculate the intervention treatment distribution in this case, we must marginalize out the natural treatment value (Young et al., 2014Young et al. (2014)). The expression above is the result of this marginalization.

B. Interventions that depends on the natural value of treatment

Note that the conditions of Corollary 1.1 are sufficient for model double robustness of the EIF but are not necessary conditions. Here, we consider model double robustness and the EIF for deterministic strategies that depend on the natural value of treatment mentioned in Section A Muñoz and Van Der Laan (2012); Haneuse and Rotnitzky (2013); Young et al. (2014); Díaz et al. (2020). These are examples of ψ^g indexed by intervention treatment distributions that do not meet the conditions of Corollary 1.1 yet model doubly robust estimators still exist.

We refer to interventions that depend on natural treatment values as 'natural treatment value interventions' (NTVI). Haneuse and Rotnitzky (2013) referred to interventions that depend on the natural value of treatment received as 'modified treatment' intervention. The cumulative probability of survival by time J under NTVIs can be identified from the extended g-formula. The extended g-formula (Robins, 1986; Young et al., 2014) for point-treatment intervention defined by $q^{g_{NTVI}}(a^+|a,\boldsymbol{l})$ is:

$$\sum_{\forall l}\sum_{\forall a}\sum_{\forall a^+} P(Y{=}1|A{=}a^+,L{=}l)q^{g_{NTVI}}(a^+|a,\boldsymbol{l})f(a|\boldsymbol{l})f(\boldsymbol{l})$$

Note that for an NTVI the exchangeability assumption for identifiability is generally more stringent than the exchangeability assumption given in Section 2. In particular, the natural value of treatment generally should not be a confounder (see Young et al., 2014 and Richardson and Robins, 2013 for more details). We consider three NTVIs:

(1) Treatment should equal what it would be in the absence of intervention provided this natural treatment value is at least δ; otherwise, it should equal δ (Taubman et al., 2009). In this intervention, $q^{g_{NTVI}}(a^+|a,\boldsymbol{l}){=}I_{\{\delta\}}(a^+)I(a{\le}\delta){+}I_{\{a\}}(a^+)I(a{>}\delta)$.

(2) For those with $\mathbf{L}{=}\boldsymbol{l}$, increase their natural treatment value by $\delta(\boldsymbol{l})$, $\forall \boldsymbol{l}$ (Muñoz and Van Der Laan, 2012). In this intervention, $q^{g_{NTVI}}(a^+|a,\boldsymbol{l}){=}I_{\{a+\delta(\boldsymbol{l})\}}(a^+)$.

(3) For those with $\mathbf{L}{=}\boldsymbol{l}$, decrease their natural treatment value by δ only if it is greater than $c(\boldsymbol{l}){+}\delta$, where $c(l){=}\min\{a\colon a{\in}\text{supp}(A) \text{ given } \mathbf{L}{=}\boldsymbol{l})\}$ (Haneuse and Rotnitzky, 2013). In this intervention, $q^{g_{NTVI}}(a^+|a,\boldsymbol{l}){=}I_{\{a-\delta\}}(a^+)I(a{>}c(\boldsymbol{l}){+}\delta){+}I_{\{a\}}(a^+)I(a{\le}c(\boldsymbol{l}){+}\delta)$.

As noted in Young et al. (2014), these NTVIs correspond to implied $q^g(a|l)$ under the generalized g-formula. Respectively, they are

(1) $q^g(a|\boldsymbol{l}){=}I_{\{\delta\}}(a)F_A(\delta|\boldsymbol{l}){+}f(a|\boldsymbol{l})I(a{>}\delta)$ (Young et al., 2014)

(2) $q^g(a|\boldsymbol{l}){=}f(a{-}\delta(\boldsymbol{l})|\boldsymbol{l})$

(3) $q^g(a|\boldsymbol{l}){=}I(a{+}\delta{>}c(\boldsymbol{l}){+}\delta)f(a{+}\delta|\boldsymbol{l}){+}I(a{\le}c(\boldsymbol{l}){+}\delta)f(a|\boldsymbol{l})$

We will use the implied intervention rules to derive EIFs using the generalized g-formula. This will be done in two steps:

Step 1: Use Theorem 1 to derive the EIFs of ψ^g in a nonparametric model that assume $F_A(\delta|\boldsymbol{l})$, $f(a{-}\delta(\boldsymbol{l})|\boldsymbol{l})$ and $f(a{+}\delta|\boldsymbol{l})$ in the equations above do not depend on the observed data law

Step 2: Add to the EIF (derived in the first step) extra term that arise when we correct for the assumption made in Step 1.

Note that in the first step, we are not assuming the observed probability of treatment is fixed and known but rather pretending that these particular functions on the treatment variable are simply some functions of $\mathbf{L}$ that do not depend on the observed data law. This procedure is valid because of the following Corollary:

Corollary B.1. *Suppose ψ^g (the parameter indexed by a choice of intervention distribution $q^g(\cdot)$) can be written as a linear combination of the form:*

$$g(\nu_1,\nu_2)=c_1\underbrace{E\{h_1(O)\}}_{\nu_1}+c_2\underbrace{E[E\{Yh_2(\boldsymbol{L})|A=a,L\}]}_{\nu_2}$$

where $h_1(O)$ is a known measurable function of O that does not depend on the observed data distribution, but $h_2(\boldsymbol{L})$ is a function of $\boldsymbol{L}$ that does depend on the observed data distribution. Then the EIF for ψ^g is given by:

$$U_{\psi^g}(O)=\underbrace{c_1h_1(O)+c_2\left[\frac{I(A=a)}{f(A|\boldsymbol{L})}h_2(\boldsymbol{L})\big[Y-E(Y|A,L)\big]+h_2(\boldsymbol{L})E(Y|A=a,L)\right]-\psi^g}_{(*)}+$$
$$\underbrace{c_2\sum_{\forall l}\sum_{\forall a}E(Y|A=a,\boldsymbol{L}=\boldsymbol{l})\frac{dh_2(\boldsymbol{L};\theta_t)}{dt}\Big|_{t=0}f(\boldsymbol{l})}_{(**)}.$$

Proof. The EIF of ν_1 equals $h_1(O)$ and the EIF of ν_2 is immediate from taking the pathwise derivative of the parameter. □

Clearly $(*)$ is the term that we obtain from Step 1) in the procedure described above, and $(**)$ is the term that we add in Step 2) to correct for our assumption made in Step 1).

Example 1. $q^g(a|\boldsymbol{l})=I_{\{\delta\}}(a)F_A(\delta|\boldsymbol{l})+f(a|\boldsymbol{l})I(a>\delta)$

The generalized g-formula is:

$$\sum_{\forall l}\sum_{\forall a}E(Y|A=a,\boldsymbol{L}=\boldsymbol{l})q^g(a|\boldsymbol{l})f(\boldsymbol{l})$$
$$=\sum_{\forall l}\sum_{\forall a}E(Y|A=a,\boldsymbol{L}=\boldsymbol{l})\{I_{\{\delta\}}(a)F_A(\delta|\boldsymbol{l})+f(a|\boldsymbol{l})I(a>\delta)\}f(\boldsymbol{l})$$

which can be equivalently written as:

$$\psi^g=E\{YI(A>\delta)\}+E\{E(Y|A=\delta,\boldsymbol{L})F_A(\delta|\boldsymbol{L})\}$$
$$=\underbrace{E\{YI(A>\delta)\}}_{\Delta_1}+\underbrace{E[E\{YF_A(\delta|\boldsymbol{L})|A=\delta,\boldsymbol{L}\}]}_{\Delta_2}$$

If we pretend that $F_A(\delta|\boldsymbol{L})$ does not depend on the observed data distribution (i.e. that it is just a function of L), then using Theorem 1, the EIFs of Δ_1 and Δ_2 in the nonparametric model are respectively $YI(A>\delta)-\Delta_1$ and

$$\frac{I_{\{\delta\}}(A)}{f(A|\boldsymbol{L})}F_A(\delta|\boldsymbol{L})\{Y-m(A,L)\}+m(\delta,\boldsymbol{L})F_A(\delta|\boldsymbol{L})-\Delta_2$$

Since $F_A(\delta|\boldsymbol{L})$ depends on the observed data distribution, we also need to consider the pathwise derivative of Δ_2 by setting all other parts of the observed data distribution except for $F_A(\delta|\boldsymbol{L};\theta)$ as fixed, i.e.

$$\sum_{\forall l}\sum_{\forall a}E(Y|A=\delta,\boldsymbol{L}=\boldsymbol{l})\frac{dF_A(\delta|\boldsymbol{l};\theta_t)}{dt}\Big|_{t=0}f(\boldsymbol{l})$$

This expression can be shown to equal $E[E\{E(Y|A=\delta,\boldsymbol{L})I(A\leq\delta)S_{A|L}|\boldsymbol{L}\}]$ where $S_{A|L}$ is the score function of A given $\boldsymbol{L}$. Therefore, we need to add to the EIF of Δ_2 the term $m(\delta,\boldsymbol{L})\{I(A\leq\delta)-F_A(\delta|\boldsymbol{L})\}$. Altogether

the EIF of ψ^g when treatment model is unknown is

$$U_{\psi^g}(O)=YI(A>\delta)+\frac{I_{\{\delta\}}(A)}{f(A|\boldsymbol{L})}F_A(\delta|\boldsymbol{L})\{Y-m(A,L)\}+m(\delta,\boldsymbol{L})I(A\leq\delta)-\psi^g$$
$$=\left\{\frac{I_{\{\delta\}}(A)}{f(A|\boldsymbol{L})}F_A(\delta|\boldsymbol{L})+I(A>\delta)\right\}\{Y-m(A,L)\}+\{m(A,L)I(A>\delta)+m(\delta,L)I(A\leq\delta)\}-\psi^g$$

The estimators obtained from EIF can be shown to be doubly robust.

Example 2. $q^g(a|\boldsymbol{l})=f(a-\delta(\boldsymbol{l})|\boldsymbol{l})$

The generalized g-formula is:

$$\sum_{\forall l}\sum_{\forall a}E(Y|A=a,\boldsymbol{L}=\boldsymbol{l})q^g(a|\boldsymbol{l})f(\boldsymbol{l})$$
$$=\sum_{\forall l}\sum_{\forall a}E(Y|A=a,\boldsymbol{L}=\boldsymbol{l})f(a-\delta(\boldsymbol{l})|\boldsymbol{l})f(\boldsymbol{l})$$
$$=\sum_{\forall a}\sum_{\forall l}E\{Yf(a-\delta(\boldsymbol{l})|\boldsymbol{l})|A=a,\boldsymbol{L}=\boldsymbol{l}\}f(\boldsymbol{l})$$

where the last line follows from Tonelli's Theorem. This can be equivalently written as:

$$\psi^g=\sum_{\forall a}\underbrace{E[E\{Yf(a-\delta(\boldsymbol{L})|\boldsymbol{L})|A=a,\boldsymbol{L}\}]}_{(*)}$$

Note that a in $(*)$ *is fixed. For example, if* $a=1$ *then* $(*)=E[E\{Yf(1-\delta(\boldsymbol{L})|\boldsymbol{L})|A=1,\boldsymbol{L}\}]$. *If we pretend that* $f(a-\delta(\boldsymbol{l})|\boldsymbol{l})$ *does not depend on the observed data distribution, then using Theorem 1, the EIF of* ψ^g *in the nonparametric model is*

$$\left[\sum_{\forall a}\frac{I_{\{a\}}(A)}{f(A|\boldsymbol{L})}\{Y-m(A,L)\}f(A-\delta(\boldsymbol{L})|\boldsymbol{L})+f(a-\delta(\boldsymbol{L})|\boldsymbol{L})m(a,L)\right]-\psi^g$$
$$=\frac{f(A-\delta(\boldsymbol{L})|\boldsymbol{L})}{f(A|\boldsymbol{L})}\{Y-m(A,L)\}+\left[\sum_{\forall a}f(a-\delta(\boldsymbol{L})|\boldsymbol{L})m(a,L)\right]-\psi^g$$

where the second equality holds because $\sum_{\forall a}I_{\{a\}}(A)=1$. *Since* $f(A-\delta(\boldsymbol{L})|\boldsymbol{L})$ *depends on the observed data distribution, we also need to consider the pathwise derivative of* ψ^g *by setting all other parts of the observed data distribution except for* $f(A-\delta(\boldsymbol{L})|\boldsymbol{L};\theta)$ *as known, i.e. Using the fact that*

$$\sum_{\forall l}\sum_{\forall a}E(Y|A=a,\boldsymbol{L}=\boldsymbol{l})\frac{df(a-\delta(\boldsymbol{l})|\boldsymbol{l};\theta_t)}{dt}\Big|_{t=0}f(\boldsymbol{l})$$

This expression can be shown to equal $E[E\{E(Y|A+\delta(\boldsymbol{L}),\boldsymbol{L})S_{A|\boldsymbol{L}}|\boldsymbol{L}\}]$. *Therefore, we need to add to the EIF of* ψ^g *the term*

$$E(Y|A+\delta(\boldsymbol{L}),\boldsymbol{L})-\sum_{\forall a}m(a+\delta(\boldsymbol{L}),\boldsymbol{L})f(a|\boldsymbol{L})=m(A+\delta(\boldsymbol{L}),\boldsymbol{L})-\sum_{\forall a}f(a-\delta(\boldsymbol{L})|\boldsymbol{L})m(a,L)$$

Altogether the EIF of ψ^g *is*

$$U_{\psi^g}(O)=\frac{f(A-\delta(\boldsymbol{L})|\boldsymbol{L})}{f(A|\boldsymbol{L})}\{Y-m(A,L)\}+m(A+\delta(\boldsymbol{L}),\boldsymbol{L})-\psi^g$$

The estimators obtained from EIF can be shown to be doubly robust.

Example 3. $q^g(a|\boldsymbol{l})=I(a+\delta>c(\boldsymbol{l})+\delta)f(a+\delta|\boldsymbol{l})+I(a\leq c(\boldsymbol{l})+\delta)f(a|\boldsymbol{l})$

The generalized g-formula is:

$$\sum_{\forall l}\sum_{\forall a} E(Y|A{=}a,\boldsymbol{L}{=}\boldsymbol{l})q^g(a|\boldsymbol{l})f(\boldsymbol{l})$$
$$=\sum_{\forall l}\sum_{\forall a} E(Y|A{=}a,\boldsymbol{L}{=}\boldsymbol{l})\{I(a{+}\delta{>}c(\boldsymbol{l}){+}\delta)f(a{+}\delta|\boldsymbol{l}){+}I(a{\le}c(\boldsymbol{l}){+}\delta)f(a|\boldsymbol{l})\}f(\boldsymbol{l})$$
$$=\sum_{\forall l}\sum_{\forall a} E(Y|A{=}a,\boldsymbol{L}{=}\boldsymbol{l})\left\{I(a{+}\delta{>}c(\boldsymbol{l}){+}\delta)\frac{f(a{+}\delta|\boldsymbol{l})f(a|\boldsymbol{l})}{f(a|\boldsymbol{l})}{+}I(a{\le}c(\boldsymbol{l}){+}\delta)f(a|\boldsymbol{l})\right\}f(\boldsymbol{l})$$

By Tonelli's Theorem, this can be equivalently written as:

$$\psi^g{=}\underbrace{E\{YI(A{\le}c(\boldsymbol{L}){+}\delta)\}}_{\Delta_1}{+}\underbrace{\sum_{\forall a}E[E\{YI(a{+}\delta{>}c(\boldsymbol{L}){+}\delta)f(a{+}\delta|\boldsymbol{L})|A{=}a,\boldsymbol{L}\}]}_{\Delta_2}$$

If we pretend that $f(a{+}\delta|\boldsymbol{L})$ does not depend on the observed data distribution, then using Theorem 1, the EIFs of Δ_1 and Δ_2 in the nonparametric model are respectively $YI(A{\le}c(\boldsymbol{L}){+}\delta)$ and

$$\left[\sum_{\forall a}I(A{+}\delta{>}c(\boldsymbol{L}){+}\delta)\frac{f(A{+}\delta|\boldsymbol{L})I_{\{a\}}(A)}{f(A|\boldsymbol{L})}\{Y{-}m(A,\boldsymbol{L})\}{+}m(a,\boldsymbol{L})I(a{+}\delta{>}c(\boldsymbol{l}){+}\delta)f(a{+}\delta|\boldsymbol{l})\right]{-}\Delta_2$$
$$=I(A{+}\delta{>}c(\boldsymbol{L}){+}\delta)\frac{f(A{+}\delta|\boldsymbol{L})}{f(A|\boldsymbol{L})}\{Y{-}m(A,\boldsymbol{L})\}{+}\sum_{\forall a}m(a{-}\delta,\boldsymbol{L})I(a{>}c(\boldsymbol{l}){+}\delta)f(a|\boldsymbol{l}){-}\Delta_2$$

Since $f(A{+}\delta|\boldsymbol{L})$ depends on the observed data distribution, we also need to consider the pathwise derivative of Δ by setting all other parts of the observed data distribution except for $f(A{+}\delta|\boldsymbol{L};\theta)$ as known, i.e.

$$\sum_{\forall l}\sum_{\forall a}E\{Y|A{=}a,\boldsymbol{L}\}I(a{+}\delta{>}c(\boldsymbol{L}){+}\delta)\frac{df(a{+}\delta|\boldsymbol{L};\theta_t)}{dt}\Big|_{t=0}f(l)$$

This expression can be shown to equal $E[E\{m(A{-}\delta,\boldsymbol{L})I(A{>}c(\boldsymbol{L}){+}\delta)S_{A|L}|\boldsymbol{L}\}]$. Therefore, we need to add to the EIF of Δ_2 the term

$$m(A{-}\delta,\boldsymbol{L})I(A{>}c(\boldsymbol{L}){+}\delta){-}E\{m(A{-}\delta,\boldsymbol{L})I(A{>}c(\boldsymbol{L}){+}\delta)|\boldsymbol{L}\}$$

Altogether the EIF of ψ^g is

$$U_{\psi^g}(O){=}YI(A{\le}c(\boldsymbol{L}){+}\delta){+}I(A{+}\delta{>}c(\boldsymbol{L}){+}\delta)\frac{f(A{+}\delta|\boldsymbol{L})}{f(A|\boldsymbol{L})}\{Y{-}m(A,\boldsymbol{L})\}{+}m(A{-}\delta,\boldsymbol{L})I(A{>}c(\boldsymbol{L}){+}\delta){-}\psi^g$$
$$=\left\{I(A{\le}c(\boldsymbol{L}){+}\delta){+}I(A{>}c(\boldsymbol{L}))\frac{f(A{+}\delta|\boldsymbol{L})}{f(A|\boldsymbol{L})}\right\}\{Y{-}m(A,\boldsymbol{L})\}{+}\{m(A{-}\delta,\boldsymbol{L})I(A{>}c(\boldsymbol{L}){+}\delta)$$
$${+}m(A,\boldsymbol{L})I(A{\le}c(\boldsymbol{L}){+}\delta)\}{-}\psi^g$$

The estimators obtained from EIF can be shown to be doubly robust.

B.1 Simulation study We describe a simulation study that shows the model double robustness of estimators under the three NTVI described previously. In particular, we focus on the first NTVI: Treatment should equal what it would be in the absence of intervention provided this natural treatment value is at least δ; otherwise, it should equal δ. This simulation study compares results from singly robust estimators (IPW and ICE) and a doubly robust estimator (weighted ICE). In weighted ICE, we carry out an outcome regression of Y conditioning on A and $\mathbf{L}$, but allowing for an observational weight defined by the NTVI. The observational weight equals $I_{\{\delta\}}(A)/f(A|\mathbf{L})F_A(\delta|\mathbf{L}){+}I(A{>}\delta)$ for this particular NTVI.

We simulated the following variables for $n{=}1000$: (L,A,Y) where L is a confounder, A is a continuous treatment and Y is the survival outcome ($Y{=}1$ if alive). In particular, $L{\sim}\text{Ber}(0.5)$, $A{\sim}\text{Pois}(5{+}3L)$ and $Y{\sim}\text{Ber}({-}3{+}0.5A{+}L)$. In our NTVI, $\delta{=}5$. The correct propensity score and outcome regression models are the ones used to generate the data. We also consider incorrectly specified propensity score and outcome regression models that assume no baseline confounder.

Table 1. Empirical SE from simulation study for the NTVI (n=1000). All values are multiplied by 100. ψ^g=0.6560.

	Bias			SE		
Estimators	All	PS only	OR only	All	PS only	OR only
IPW	0.01	0.01	-1.94	2.37	2.37	2.11
ICE	-0.04	-5.00	-0.04	1.60	1.55	1.60
Weighted ICE	0.00	0.01	-0.01	2.10	2.25	2.04

Table 1 shows the bias and standard error from the NTVI. Theory indicates that weighted ICE is doubly robust such that if the propensity score model or the outcome regression model is correctly specified, it will be consistent. Our simulation results agree with this theory. Interestingly, weighted ICE is at least as efficient as the IPW estimator, although this is not supported by theory.

C. Proof of Theorem 1 and Corollary 1.1

Proof. Proof for Theorem 1: The efficient influence function in the nonparametric model $\mathcal{F}_{NP}$ is defined as the unique mean zero, finite variance random variable such that

$$\frac{d\psi^g(\theta_t)}{dt}\Big|_{t=0}=E\{U_{\psi^g}(O)S(O)\}$$

where $d\psi^g(\theta_t)/dt|_{t=0}$ is known as the pathwise derivative of parameter ψ^g along a parametric submodel indexed by t, and S_O is the score function of the parametric submodel evaluated at $t{=}0$. We aim to derive $d\psi^g(\theta_t)/dt|_{t=0}$ for the parameter defined by the

$$\psi^g=g(\nu_1,\nu_2)=c_1\underbrace{E\{h_1(O)\}}_{\nu_1}+c_2\underbrace{E[E\{h_2(O)|A=a,L\}]}_{\nu_2}$$

For notational simplicity, we let $S_O\equiv S_O(O)$. Moreover in the expressions below, $S_{A|B,C}\equiv S_{A|B,C}(A,B,C)$. Taking this pathwise derivative of parameter we obtain

$$
\begin{aligned}
&d\psi^g(\theta_t)/dt|_{t=0}\\
&=c_1d\nu_1(\theta_t)/dt|_{t=0}+c_2d\nu_2(\theta_t)/dt|_{t=0}\ \text{(by differentiation rule)}\\
&=c_1E\Big[\big\{h_1(O)-\nu_1\big\}S_O\Big]+c_2\Big[E\{E(h_2(O)S_{Y|A=a,\mathbf{L}}|A=a,\mathbf{L})\}+E\{E(h_2(O)|A=a,\mathbf{L})S_L\}\Big]\\
&=c_1E\Big[\big\{h_1(O)-\nu_1\big\}S_O\Big]+c_2\left(E\Big[E\Big\{\big(h_2(O)-E(h_2(O)|A=a,\mathbf{L})\big)S_{Y|A=a,\mathbf{L}}|A=a,\mathbf{L}\Big\}\Big]+E\Big[\big\{E(h_2(O)|A=a,\mathbf{L})-\nu_2\big\}S_O\Big]\right)\\
&=c_1E\Big[\big\{h_1(O)-\nu_1\big\}S_O\Big]+c_2\left(E\left[\frac{I(A=a)\big\{h_2(O)-E(h_2(O)|A=a,\mathbf{L})\big\}}{f(A|\mathbf{L})}S_{Y|A,\mathbf{L}}\right]+E\Big[\big\{E(h_2(O)|A=a,\mathbf{L})-\nu_2\big\}S_O\Big]\right)\\
&=c_1E\Big[\big\{h_1(O)-\nu_1\big\}S_O\Big]+c_2\left(E\left[\frac{I(A=a)\big\{h_2(O)-E(h_2(O)|A=a,\mathbf{L})\big\}}{f(A|\mathbf{L})}S_O\right]+E\Big[\big\{E(h_2(O)|A=a,\mathbf{L})-\nu_2\big\}S_O\Big]\right)
\end{aligned}
$$

Hence, we arrive at the EIF:

$$U_{\psi^g}(O)=c_1E\Big[\big\{h_1(O)-\nu_1\big\}S_O\Big]+c_2\left[\frac{I(A=a)}{f(A|\mathbf{L})}\left[h_2(O)-E\{h_2(O)|A,\mathbf{L}\}\right]+E\{h_2(O)|A=a,\mathbf{L}\}\right]-g(\nu_1,\nu_2).$$

□

Proof. Proof for Corollary 1.1:

Suppose that the estimates for $E(Y|A,L)$ and $f(A|L)$ are denoted by $E(Y|A,L;\hat{\theta})$ and $f(A|L;\hat{\alpha})$, respectively. Here, $E(Y|A,L;\hat{\theta})$ is parameterized by θ and $f(A|L;\hat{\alpha})$ by α, and estimates of θ and α are obtained using some estimation method (e.g. maximum likelihood). Let ψ^{g^*}, θ^* and α^* denote probability limits of $\hat{\psi^g}$ $\hat{\theta}$ and $\hat{\alpha}$. To prove that an estimator derived from and EIF is doubly robust, it suffices to show that following the estimating equations are unbiased:

$$\mathbb{P}_n\left(c_1h_1(O)+c_2\left[\frac{I(A=a)}{f(A|\mathbf{L};\hat{\alpha})}\left[h_2(O)-E\{h_2(O)|A,L;\hat{\theta}\}\right]+E\{h_2(O)|A=a,L;\hat{\theta}\}\right]-\hat{\psi^g}\right)$$

First, suppose the treatment model is correctly specified such that $f(A|L;\alpha^*)=f(A|L)$:

$$
\begin{aligned}
&E\left(c_1h_1(O)+c_2\left[\frac{I(A=a)}{f(A|\mathbf{L};\alpha^*)}\left[h_2(O)-E\{h_2(O)|A,L;\theta^*\}\right]+E\{h_2(O)|A=a,L;\theta^*\}\right]-\psi^{g^*}\right)\\
&=E\left(c_1h_1(O)+c_2\left[\underbrace{\frac{f(A|\mathbf{L})}{f(A|\mathbf{L};\alpha^*)}}_{(a)}\left[\underbrace{E\{h_2(O)|A,L\}-E\{h_2(O)|A,L;\theta^*\}}_{(b)}\right]+E\{h_2(O)|A=a,L;\theta^*\}\right]-\psi^{g^*}\right)\\
&=E\left[c_1h_1(O)+c_2E\{h_2(O)|A=a,L\}-\psi^{g^*}\right] \qquad \text{(C.1)}\\
&=0 \text{ (when } \psi^{g^*}=\psi^g)
\end{aligned}
$$

where Line (C.1) holds because Expression (a) equals 1. Next, suppose that the outcome regression model is correctly specified. By the condition given in the Corollary, $E\{h_2(O)|A,L;\theta^*\}=\tilde{h}_2(A,L)E(Y|A,L;\theta^*)=E\{h_2(O)|A,L\}$, and therefore Line (C.1) holds because Expression (b) equals 0. □

C.1 More examples of Theorem 1 We now consider some applications of Theorem 1 and Corollary 1.1 to examples where the intervention distribution indexing ψ^g depends on the observed treatment process.

Example 1. *Consider a variation of the grace period treatment initiation strategies defined in Section A for $J=1$, $m=0$, such that, rather than withholding treatment when $L^*=0$, no intervention is made. The intervention treatment distribution is then given by $q^g(a|\boldsymbol{l})=(1-l^*)f(a|\boldsymbol{l})+l^*a$.*

For this choice of intervention distribution we have:

$$
\begin{aligned}
\psi^g&=E_{\mathbf{L}}\left\{\sum_{a=0}^{1}E(Y|a,\mathbf{L})q^g(a|\mathbf{L})\right\}\\
&=E_{\mathbf{L}}\left[\sum_{a=0}^{1}E(Y|a,\mathbf{L})\left\{(1-L^*)f(a|\mathbf{L})+I_{\{1\}}(a)L^*\right\}\right]\\
&=E_{\mathbf{L},A}\left[E\left\{Y(1-L^*)|A,\mathbf{L}\right\}\right]+E_{\mathbf{L}}\left[E\left\{YL^*|A=1,\mathbf{L}\right\}\right].
\end{aligned}
$$

Selecting $a^*=1$, $c_1=c_2=1$, $h_1(O)=Y(1-L^*)$, and $h_2(O)=YL^*$, we have

$$
\begin{aligned}
\psi^g&=c_1E\{h_1(O)\}+c_2E[E\{h_2(O)|A=a^*,\mathbf{L}\}]\\
&=\underbrace{E\{Y(1-L^*)\}}_{\nu_1}+\underbrace{E[E\{YL^*|A=1,L\}]}_{\nu_2}
\end{aligned}
$$

by Theorem 1 and further, the EIF for ψ^g is given by

$$U_{\psi^g}(O)=Y(1-L^*)+\frac{AL^*}{f(A|\mathbf{L})}\left\{Y-m(A,\mathbf{L})\right\}+m(1,\mathbf{L})L^*-\psi^g.$$

This can be re-expressed as

$$U_{\psi^g}(O)=\frac{q^g(A|\mathbf{L})}{f(A|\mathbf{L})}\left\{Y-m(A,\mathbf{L})\right\}+m(A,\mathbf{L})(1-L^*)+m(1,\mathbf{L})L^*-\psi^g$$

which is a useful representation for deriving doubly robust estimators. By Corollary 1.1, estimators based on the EIF for ψ^g in this case will be doubly robust because ν_1 can be estimated non-parametrically, and EIF-based estimators for ν_2 are doubly robust. In particular, these will be consistent if either $f(A|\mathbf{L})$ or $m(A,\mathbf{L})$ is consistently estimated, not necessarily both.

Example 2. *Representative interventions for $J=1$. The intervention treatment distribution is given by $q^g(a|\boldsymbol{l})=f(a|\boldsymbol{l},R=1)$ where $R=I(A\geq\delta)$.*

In this case we have

$$\psi^g = E_{\mathbf{L}}\left\{\sum\nolimits_{a=0}^{1} E(Y|a,\mathbf{L})q^g(a|\mathbf{L})\right\} = E_{\mathbf{L}}\left[E_A\{E(Y|A,\mathbf{L},R=1)|\mathbf{L},R=1\}\right] = E_{\mathbf{L}}\{E(Y|\mathbf{L},R=1)|\}.$$

Following previous results Young et al. (2019), we can see that, for this choice of intervention treatment distribution and $J=1$, ψ^g is only a function of R, a coarsening of A, and takes the same form as the g-formula indexed by the static deterministic strategy that sets treatment to 1 but with R playing the role of treatment. We can apply Theorem 1 and Corollary 1.1 replacing the treatment A with R and a^* with r^*. Specifically, selecting $r^*=1$, $c_2=1$, $h_1(O)=0$, $h_2(O)=Y$, by Theorem 1 we have

$$\psi^g = c_2 E[E\{h_2(O)|R=r^*,L\}] = \underbrace{E\{E(Y|R=1,\mathbf{L})\}}_{\nu_2}$$

and the EIF for ψ^g is

$$U_{\psi^g}(O) = \frac{I(R=1)}{f(R|\mathbf{L})}\{Y - m(R,\mathbf{L})\} + m(1,\mathbf{L}) - \psi^g.$$

By Corollary 1.1, estimators based on the EIF will be model doubly robust, i.e., consistent if either models for $f(R|L)$ *or* $E(Y|R,L)$ are correctly specified.

C.2 Examples for longitudinal stochastic treatment strategy ($J=2$) We now give an extension of Theorem 1 for the case where $J=2$:

Corollary C.1. *For $J=2$, if ψ^g can be expressed as:*

$$c_1\underbrace{E\{h_1(O)\}}_{\nu_1} + c_2\underbrace{E[E\{h_2(O)|A_0=a_{0,2},\boldsymbol{L}_0\}]}_{\nu_2} + \tag{C.2}$$
$$c_3\underbrace{E[E\{h_3(O)|A_1=a_{1,3},Y_1,\bar{\boldsymbol{L}}_1,A_0\}]}_{\nu_3} + c_4\underbrace{E(E[E\{h_4(O)|\bar{A}_1=\bar{a}_{1,4},Y_1,\bar{\boldsymbol{L}}_1\}|A_0=a_{0,4},\boldsymbol{L}_0])}_{\nu_4}$$

where $h_j(O)$ are known measurable functions of the observed data ($j=1,2,3,4$). Then, the efficient influence function is given as

$$U_{\psi^g}(O) = c_1U_{\nu_1}(O) + c_1U_{\nu_2}(O) + c_3U_{\nu_3}(O) + c_4U_{\nu_4}(O) - \psi^g$$

where U_{ν_j} is the EIF of individual parameters ν_j ($j=1,2,3,4$).

Example 1. *Consider a stochastic dynamic treatment strategies that allow for a one-month grace period during which initiation of a treatment is required. In particular, this treatment strategy requires one to initiate treatment within 1 month after the first time that L_j (e.g. AIDS status) becomes 1 ($L_j \in \boldsymbol{L}_j$). It is assumed that once $L_j=1$ then $L_{j+1}=1$. Table 2 shows the possible observations under this treatment strategy. The intervention distribution $q^g(a_j|1,\bar{\boldsymbol{l}}_j,\bar{a}_{j-1})$ equals $I_(a_0=0)I(L_0=0)+f(a_0|\boldsymbol{l}_0)I(L_0=1)$ at time $j=0$ and $I(a_1=0)I(\bar{L}_1=0)+I(a_j=1)I(L_0=1)+f(a_1|1,\bar{\boldsymbol{l}}_1,a_0)I(L_0=0,L_1=1)$ at time $j=1$.*

Table 2. Possible observations under the one-month grace period treatment strategy.

j	A_j	L_j^*
0	0/1	1
1	1	1
0	0	0
1	0/1	1
0	0	0
1	0	0

The g-formula for this intervention is given by

$$\psi^g = E_{f_{\mathbf{L}_0}}\left\{E_{q^g_{A_0}}\left(E_{f_{\mathbf{L}_1,Y_1}}\left[E_{q^g_{A_1}}\left\{E_{f_{Y_2}}(Y_2|\bar{A}_1,\bar{\mathbf{L}}_1,Y_1)|A_0,\bar{\mathbf{L}}_1,Y_1\right\}|A_0,\mathbf{L}_0\right]|\mathbf{L}_0\right)\right\}$$

$$= E_{f_{\mathbf{L}_0}}\left[E_{f_{\mathbf{L}_1,A_1,\bar{Y}_2}}\{Y_2(1-L_0)L_1|A_0=0,\mathbf{L}_0\}\right]+$$

$$E_{f_{\bar{\mathbf{L}}_1,A_0,Y_1}}\left[E_{f_{Y_2}}\{Y_2L_0L_1|A_1=1,A_0,\bar{\mathbf{L}}_1\}\right]+$$

$$E_{f_{\mathbf{L}_0}}\left(E_{f_{\mathbf{L}_1,Y_1}}\left[E_{f_{Y_2}}\{Y_2(1-L_0)(1-L_1)|\bar{A}_1=0,\bar{\mathbf{L}}_1,Y_1\}|\mathbf{L}_0,A_0=0\right]\right)$$

By Expression (C.2), $c_2=c_3=c_4=1$, $h_1(O)=0$, $h_2(O)=Y_2(1-L_0)L_1$, $h_3(O)=Y_2L_0L_1$, $h_4(O)=Y_2(1-L_0)(1-L_1)$, and therefore

$$\psi^g = c_2E[E\{h_2(O)|A_0=0,\mathbf{L}_0\}]+c_3E[E\{h_3(O)|A_1=1,Y_1,\bar{\mathbf{L}}_1,A_0\}]+$$

$$c_4E(E[E\{h_4(O)|\bar{A}_1=0,Y_1,\bar{\mathbf{L}}_1\}|A_0=0,\mathbf{L}_0])$$

$$=\underbrace{E\left[E\{Y_2(1-L_0)L_1|A_0=0,\mathbf{L}_0\}\right]}_{\nu_2}+ \tag{C.3}$$

$$\underbrace{E\left[E\{Y_2L_0L_1|A_1=1,A_0,\bar{\mathbf{L}}_1\}\right]}_{\nu_3}+ \tag{C.4}$$

$$\underbrace{E\Big(E\left[E\{Y_2(1-L_0)(1-L_1)|\bar{A}_1=0,\bar{\mathbf{L}}_1,Y_1\}|\mathbf{L}_0,A_0=0\right]\Big)}_{\nu_4} \tag{C.5}$$

Expressions (C.3)–(C.5) correspond to a sum of three static treatment strategies:

(1) ν_2 in Expression (C.3) implies the following static treatment strategy: given observed data $O=(\mathbf{L}_0,A_0,W)$ where $W=(\mathbf{L}_1,A_1,\bar{Y}_2)$ and outcome $h_2(O)$, treat all individuals at time 0. The EIF for ν_2 obtained after substituting Y in Expression (7) with $h_2(O)$ is

$$\frac{(1-A_0)(1-L_0)}{f(A_0|\mathbf{L}_0)}\left[Y_2L_1-E\{Y_2L_1|A_0=0,\mathbf{L}_0\}\right]+(1-L_0)E\{Y_2L_1|A_0=0,\mathbf{L}_0\}-\nu_2$$

(2) ν_3 in Expression (C.4) implies the following static treatment strategy: given observed data $O=(\mathbf{V},A_1,Y_2)$ where $\mathbf{V}=(\mathbf{L}_0,A_0,\mathbf{L}_0,Y_1)$ and outcome $h_3(O)$, treat all individuals at time 1. The EIF for ν_3 obtained after substituting Y in Expression (7) with $h_3(O)$ is

$$\frac{A_1(1-L_0)L_1}{f(A_1|Y_1=1,\bar{\mathbf{L}}_1,A_0)}(Y_2-Q_1)+(1-L_0)L_1Q_1^{A_1=1}-\nu_3$$

(3) ν_4 in Expressions (C.5) implies the following static treatment strategy: given observed data $O=(\mathbf{L}_0,A_0,Y_1,\mathbf{L}_1,Y_2)$ and outcome $h_4(O)$, never treat for all individuals at times 0 and 1. The EIF for ν_4 obtained after substituting Y_2 in Expression (C.12) (when $J=2$) with $h_4(O)$ is

$$(Y_2-Q_1)\left\{\frac{(1-A_0)(1-A_1)(1-L_0)(1-L_1)}{f(A_1|Y_1=1,\bar{\mathbf{L}}_1,A_0)f(A_0|\mathbf{L}_0)}\right\}+\frac{(1-A_0)(1-L_0)}{f(A_0|\mathbf{L}_0)}\left\{Q_1^{A_1=0}-E\{Q_1^{A_1=0}(1-L_0)(1-L_1)|\mathbf{L}_0,A_0)\right\}$$
$$+(1-L_0)E\{Q_1^{A_1=0}(1-L_1)|\mathbf{L}_0,A_0=0)-\nu_4$$

After some algebra and realizing that

$$Q_0=(1-L_0)E\{Q_1^{A_1=0}(1-L_1)+Y_2L_1|\mathbf{L}_0,A_0\}$$

it can be shown that the EIF for ψ^g equals

$$
\begin{aligned}
&(Y_2-Q_1)\left\{\frac{(1-A_0)(1-A_1)(1-L_0)(1-L_1)}{f(A_1|Y_1=1,\bar{\mathbf{L}}_1,A_0)f(A_0|\mathbf{L}_0)}+\frac{A_1L_0L_1}{f(A_1|\bar{\mathbf{L}}_1,A_0,Y_1=1)}\right\}+\\
&Y_2(1-L_0)L_1\frac{(1-A_0)}{f(A_0|\mathbf{L}_0)}+Q_1^{A_1=0}(1-L_0)(1-L_1)\frac{(1-A_0)}{f(A_0|\mathbf{L}_0)}+Q_1^{A_1=1}L_0L_1+ \qquad \text{(C.6)}\\
&Q_0^{A_0=0}(1-L_0)-Q_0(1-L_0)\frac{(1-A_0)}{f(A_0|\mathbf{L}_0)}-\psi^g
\end{aligned}
$$

After some algebra, Expression (C.6) can be shown to equal

$$
\begin{aligned}
&(Y_2-Q_1)\frac{\prod_{j=0}^{1}q^g(A_j|Y_j=1,\bar{\mathbf{L}}_j,\bar{A}_{j-1})}{\prod_{j=0}^{1}f(A_j|Y_j=1,\bar{\mathbf{L}}_j,\bar{A}_{j-1})}+\\
&\frac{q^g(A_0|\mathbf{L}_0)}{f(A_0|\mathbf{L}_0)}\left\{Q_1(1-L_0)L_1+Q_1^{A_1=0}(1-L_0)(1-L_1)+Q_1^{A_1=1}L_0L_1-Q_0\right\}+\\
&Q_0L_0+Q_0^{A_0=0}(1-L_0)-\psi^g
\end{aligned}
$$

which is consistent with the results derived from taking the pathwise derivative of the target parameter ψ^g.

We also note the following

- The EIF-based estimators for Expression (C.5) will be consistent as long as (1) the models for $f(A_0|\mathbf{L}_0)$ and $f(A_1|Y_1=1,\bar{\mathbf{L}}_1,A_0)$ are correctly specified, OR (2) the models for Q_1 and Q_0 are correctly specified, OR (3) the model for Q_1 is correctly specified and the model for $f(A_0|\mathbf{L}_0)$ is correctly specified.
- The EIF-based estimators for Expression (C.4) will be consistent as long as (1) the model for $f(A_1|Y_1=1,\bar{\mathbf{L}}_1,A_0)$ is correctly specified OR (2) the model for Q_1 is correctly specified.
- The EIF-based estimators for Expression (C.3) will be consistent as long as (1) the model for $f(A_0|\mathbf{L}_0)$ is correctly specified OR (2) the model for Q_0 is correctly specified.

Hence the EIF-based estimators will be $J+1$ model multiply robust.

Example 2. *Consider our proposed incremental propensity score intervention for two time points. As before, for $L_j^*\in\boldsymbol{L}_j$, decrease the probability of non-treatment to $P(A_j=0|\bar{\boldsymbol{L}}_j,\bar{A}_{j-1},Y_j=1)\delta$ (for $\delta\in[0,1]$) if $L_j^*=1$, otherwise do not intervene if $L_j^*=0$.*

The g-formula for this intervention for $J=2$ is

$$
\begin{aligned}
\psi^g=&E_{f_{L_0}}\left\{E_{q^g_{A_0}}\left(E_{f_{L_1,Y_1}}\left[E_{q^g_{A_1}}\left\{E_{f_{Y_2}}(Y_2|\bar{A}_1,\bar{\boldsymbol{L}}_1,Y_1)|A_0,\bar{\boldsymbol{L}}_1,Y_1\right\}|A_0,\boldsymbol{L}_0\right]|\boldsymbol{L}_0\right)\right\}\\
=&E_{f_{\bar{L}_1,\bar{A}_1,\bar{Y}_2}}\left\{Y_2(L_1^*\delta+1-L_1^*)(L_0^*\delta+1-L_0^*)\right\}+\\
&E_{f_{L_0}}\left[E_{f_{L_1,A_1,\bar{Y}_2}}\left\{Y_2(L_1^*\delta+1-L_1^*)L_0^*(1-\delta)|\boldsymbol{L}_0,A_0=1\right\}\right]+\\
&E_{f_{\bar{L}_1,A_0,Y_1}}\left[E_{f_{Y_2}}\left\{Y_2L_1^*(1-\delta)(L_0^*\delta+1-L_0^*)|\bar{\boldsymbol{L}}_1,A_1=1,A_0,Y_1\right\}\right]+\\
&E_{f_{L_0}}\left(E_{f_{L_1,Y_1}}\left[E_{f_{Y_2}}\left\{Y_2L_1^*(1-\delta)L_0^*(1-\delta)|\bar{A}_1=1,\bar{\boldsymbol{L}}_1,Y_1\right\}|A_0=1,L_0\right]\right)
\end{aligned}
$$

By Expression (C.2), $c_1=1$, $c_2=(1-\delta)$, $c_3=(1-\delta)$, $c_4=(1-\delta)^2$, $h_1(O)=Y_2(L_1^*\delta+1-L_1^*)(L_0^*\delta+1-L_0^*)$, $h_2(O)=Y_2(L_1^*\delta+1-L_1^*)L_0^*(1-\delta)$, $h_3(O)=Y_2L_1^*(L_0^*\delta+1-L_0^*)$, $h_4(O)=Y_2L_1^*L_0^*$, *and therefore*

$$\psi^g=c_1E\{h_1(O)\}+c_2E[E\{h_2(O)|A_0=1,\boldsymbol{L}_0\}]+ c_3E[E\{h_3(O)|A_1=1,Y_1,\bar{\boldsymbol{L}}_1,A_0\}]+c_4E(E[E\{h_4(O)|\bar{A}_1=1,Y_1,\bar{\boldsymbol{L}}_1\}|A_0=1,\boldsymbol{L}_0])$$

$$=\underbrace{E\left\{Y_2(L_1^*\delta+1-L_1^*)(L_0^*\delta+1-L_0^*)\right\}}_{\nu_1}+ \tag{C.7}$$

$$(1-\delta)\underbrace{E\Big[E\left\{Y_2(L_1^*\delta+1-L_1^*)L_0^*|\boldsymbol{L}_0,A_0=1\right\}\Big]}_{\nu_2}+ \tag{C.8}$$

$$(1-\delta)\underbrace{E\Big[E\left\{Y_2L_1^*(L_0^*\delta+1-L_0^*)|\bar{\boldsymbol{L}}_1,A_1=1,A_0,Y_1\right\}\Big]}_{\nu_3}+ \tag{C.9}$$

$$(1-\delta)^2\underbrace{E\bigg(E\Big[E\left\{Y_2L_1^*L_0^*|\bar{A}_1=1,\bar{\boldsymbol{L}}_1,Y_1\right\}|A_0=1,L_0\Big]\bigg)}_{\nu_4} \tag{C.10}$$

Let

$$Q_1=E_{f_{Y_2}}(Y_2|\bar{A}_1,\bar{\boldsymbol{L}}_1,Y_1);\quad Q_1^{A_1=a_1}=E_{f_{Y_2}}(Y_2|A_1=a_1,A_0,\bar{\boldsymbol{L}}_1,Y_1)\ \textit{for}\ a_1=0{,}1$$

$$Q_0=E_{f_{L_1,Y_1}}\Big[E_{q^g_{A_1}}\left\{Q_1|A_0,\bar{\boldsymbol{L}}_1,Y_1\right\}|A_0,\boldsymbol{L}_0\Big];$$

$$Q_0^{A_0=a_0}=E_{f_{L_1,Y_1}}\Big[E_{q^g_{A_1}}\left\{Q_1|A_0,\bar{\boldsymbol{L}}_1,Y_1\right\}|A_0=a_0,\boldsymbol{L}_0\Big]\ \textit{for}\ a_0=0{,}1$$

The EIF of ν_1 *in Expression (C.7) is simply* $Y_2(L_1^*\delta+1-L_1^*)(L_0^*\delta+1-L_0^*)-\nu_1$. ν_2, ν_3 *and* ν_4 *in Expressions (C.8)–(C.10) respectively imply static treatment strategies on outcomes defined by* $h_2(O)$, $h_3(O)$ *and* $h_4(O)$. *More specifically:*

(1) ν_2 *in Expression (C.8) implies the following static treatment strategy: given observed data* $O=(\boldsymbol{L}_0,A_0,W)$ *where* $W=(Y_1,\boldsymbol{L}_1,A_1,Y_2)$ *and outcome* $h_2(O)$, *treat all individuals at time 0. After replacing* Y *in Expression (7) with* $h_2(O)$, *the EIF for* ν_2 *is*

$$\frac{A_0L_0^*}{f(A_0|\boldsymbol{L}_0)}\left[Y_2(L_1^*\delta+1-L_1^*)-E\{Y_2(L_1^*\delta+1-L_1^*)|A_0=1,\boldsymbol{L}\}\right]+E\{Y_2(L_1^*\delta+1-L_1^*)L_0^*|A_0=1,\boldsymbol{L}_0\}-\nu_2$$

(2) ν_3 *in Expression (C.9) implies the following static treatment strategy: given observed data* $O=(\boldsymbol{V},A_1,Y_2)$ *where* $\boldsymbol{V}=(\boldsymbol{L}_0,A_0,\boldsymbol{L}_1,Y_1)$ *and outcome* $h_3(O)$, *treat all individuals at time 1. After replacing* Y *in Expression (7) with* $h_3(O)$, *the EIF for* ν_3 *is*

$$\frac{A_1L_1^*(L_0^*\delta+1-L_0^*)}{f(A_1|Y_1=1,A_0,\bar{\boldsymbol{L}}_1)}(Y_2-Q_1)+L_1^*(L_0^*\delta+1-L_0^*)Q_1^{A_1=1}-\nu_3$$

(3) ν_4 *in Expression (C.10) implies the following deterministic treatment strategy: given observed data is* $O=(\boldsymbol{L}_0,A_0,Y_1,\boldsymbol{L}_1,A_1,Y_2)$ *and outcome* $h_4(O)$, *treat all individuals at time 0 and 1. After replacing* Y_2 *in Expression (C.12) (when* $J=2$*) with* $h_2(O)$, *the EIF for* ν_4 *is therefore*

$$\frac{L_1^*L_0^*\bar{A}_1}{\prod_{j=0}^1 f(A_j|Y_1=1,\bar{\boldsymbol{L}}_j,\bar{A}_{j-1})}(Y_2-Q_1)+\frac{A_0L_0^*}{f(A_0|\boldsymbol{L}_0)}\Big[Q_1^{A_1=1}L_1^*-E\Big\{Q_1^{A_1=1}L_1^*|A_0,L_0\Big\}\Big]$$
$$+E\Big\{Q_1^{A_1=1}L_1^*|A_0=1,L_0\Big\}L_0^*-\nu_4$$

After some algebra and realizing that

$$Q_0 = E\Big[Q_1(L_1^*\delta+1-L_1^*)+Q_1^{A_1=1}L_1^*(1-\delta)\,|\,A_0,L_0\Big]$$
$$= E\Big[Y_2(L_1^*\delta+1-L_1^*)+Q_1^{A_1=1}L_1^*(1-\delta)\,|\,A_0,L_0\Big]$$

it can be shown that the EIF for ψ^g equals:

$$\begin{aligned}
&(Y_2-Q_1)\left\{\frac{L_1^*A_1(1-\delta)(L_0^*\delta+1-L_0^*)}{f(A_1|Y_1=1,A_0,\bar{\boldsymbol{L}}_1)}+\frac{L_1^*A_1(1-\delta)L_0^*A_0(1-\delta)}{f(A_1|Y_1=1,A_0,\bar{\boldsymbol{L}}_1)f(A_0|\boldsymbol{L}_0)}\right\}+\\
&Y_2(L_1^*\delta+1-L_1^*)(L_0^*\delta+1-L_0^*)+Y_2(L_1^*\delta+1-L_1^*)\frac{L_0^*A_0(1-\delta)}{f(A_0|L_0)}+\\
&(L_0^*\delta+1-L_0^*)Q_1^{A_1=1}L_1^*(1-\delta)+\frac{L_0^*A_0(1-\delta)}{f(A_0|L_0)}\{Q_1^{A_1=1}L_1^*(1-\delta)-Q_0\}+Q_0^{A_0=1}L_0^*(1-\delta)-\psi^g
\end{aligned}\tag{C.11}$$

After some algebra, Expression (C.11) can be shown to equal

$$\begin{aligned}
U_{\psi^g}(O)=(Y_2-Q_1)&\frac{\prod_{j=0}^1 q^g(A_j|Y_j=1,\bar{\boldsymbol{L}}_j,\bar{A}_{j-1})}{\prod_{j=0}^1 f(A_j|Y_j=1,\bar{\boldsymbol{L}}_j,\bar{A}_{j-1})}+\frac{q^g(A_0|\boldsymbol{L}_0)}{f(A_0|\boldsymbol{L}_0)}\{Q_1(L_1^*\delta+1-L_1^*)+Q_1^{A_1=1}L_1^*(1-\delta)-Q_0\}+\\
&Q_0(L_0^*\delta+1-L_0^*)+Q_0^{A_0=1}L_0^*(1-\delta)-\psi^g
\end{aligned}$$

which is consistent with the results derived from taking the pathwise derivative of the target parameter ψ^g.

We also note the following

- The EIF-based estimators for ν_2 will be consistent as long as (1) the model for $f(A_0|\mathbf{L}_0)$ is correctly specified OR (2) the models Q_0 is correctly specified.
- The EIF-based estimators for ν_3 will be consistent as long as (1) the model for $f(A_1|Y_1=1,\bar{\mathbf{L}}_1,A_0)$ is correctly specified OR (2) the models Q_1 is correctly specified.
- The EIF-based estimators for ν_4 will be consistent as long as (1) the models for $f(A_1|Y_1=1,\bar{\mathbf{L}}_1,A_0)$ and $f(A_0|\mathbf{L}_0)$ are correctly specified, OR (2) the models for Q_1 and Q_0 are correctly specified, OR (3) the model for Q_1 is correctly specified and the model for $f(A_0|\mathbf{L}_0)$ is correctly specified.

Hence the EIF-based estimators will be $J+1$ model multiply robust. Note that there are EIF-based estimators that are even more robust to model misspecification (e.g. some can be 2^J multiply robust), but these estimators are out of the scope of this paper. Next, we consider another stochastic dynamic treatment strategy that allow for a grace period during which initiation of a treatment is required.

Example 3. *Consider the representative intervention given in Section 5 for $J=2$. The intervention distribution is given by $q^g(a_j|1,\bar{\boldsymbol{l}}_j,\bar{a}_{j-1})=f(a_j|1,\bar{\boldsymbol{l}}_j,\bar{a}_{j-1},a_j\geq\delta)$. For a static treatment strategy where treatment is forced to take values $\bar{a}_{J-1}$, the g-formula corresponds to (C.2) where $h_1(O)=h_2(O)=h_3(O)=0$, $h_4(O)=Y_2$ and $c_4=1$. The EIF of parameter ψ^g is thus given by*

$$\left\{\sum_{j=0}^{J-1}Y_j\frac{I(\bar{A}_j=\bar{a}_j)}{\prod_{k=1}^j f(A_k|Y_k=1,\bar{A}_{k-1},\bar{\boldsymbol{L}}_k)}(T_{j+1}-T_j)+T_0\right\}-\psi^g \tag{C.12}$$

where $T_J=Y_J$, and iteratively from $j=J-1,\ldots,0$, $T_j\equiv T_j(\bar{L}_j,\bar{Y}_j,\bar{A}_j=\bar{a}_j)=E\{T_{j+1}|\bar{\boldsymbol{L}}_j,\bar{A}_j=\bar{a}_j,Y_j=1\}$ if $Y_j=1$ and $T_j=0$ if $Y_j=0$. The g-formula under a representative intervention can be shown to equal to the g-formula for static treatment intervention where A_j is replaced with R_j at all time points (see Young et al., 2019 for more details).

D. Derivation of the EIF for the class of intervention distributions defined in Theorem 2

Proof. The efficient influence function in the nonparametric model $\mathcal{F}_{NP}$ is defined as the unique mean zero, finite variance random variable such that

$$\frac{d\psi^g(\theta_t)}{dt}\Big|_{t=0} = E\{U_{\psi^g}(O)S(O)\}$$

where $d\psi^g(\theta_t)/dt|_{t=0}$ is known as the pathwise derivative of parameter ψ^g along a parametric submodel indexed by t, and S_O is the score function of the parametric submodel evaluated at $t=0$. We aim to derive $d\psi^g(\theta_t)/dt|_{t=0}$ for our parameter defined by the class of intervention distribution defined by

$$\begin{aligned} &q^g(a_j|1,\bar{\boldsymbol{l}}_j,\bar{a}_{j-1}) \\ &\quad = c_1 I_{\{a_j^*\}}(a_j)h_1(\bar{\boldsymbol{l}}_j,a_{j-1}) + c_2 f(a_j|1,\bar{\boldsymbol{l}}_j,\bar{a}_{j-1})h_2(\bar{\boldsymbol{l}}_j,a_{j-1}) + \\ &\quad c_{3,a_j} p^*(a_j|1,\bar{\boldsymbol{l}}_j,\bar{a}_{j-1})h_{3,a_j}(\bar{\boldsymbol{l}}_j,a_{j-1}) \end{aligned}$$

For notational simplicity, we let $S_O \equiv S_O(O)$. Moreover in the expressions below, $S_{A|B,C} \equiv S_{A|B,C}(A,B,C)$. Taking this pathwise derivative of parameter we obtain

$$d\psi^g(\theta_t)/dt|_{t=0} =$$

$$E_{f_{\mathbf{L}_0}}\left(...E_{f_{\mathbf{L}_{J-1},Y_{J-1}}}\left[E_{q^g_{A_{J-1}}}\left\{E_{f_{Y_J}}(Y_J S_{Y_J|\bar{\mathbf{L}}_{J-1},\bar{A}_{J-1},\bar{Y}_{J-1}}|\bar{\mathbf{L}}_{J-1},\bar{A}_{J-1},\bar{Y}_{J-1})|\bar{\mathbf{L}}_{J-1},\bar{A}_{J-2},\bar{Y}_{J-1}\right\}|\bar{\mathbf{L}}_{J-2},\bar{A}_{J-2},\bar{Y}_{J-2}\right]...\right)+ \tag{D.13}$$

$$\sum_{j=1}^{J-1} E_{f_{\mathbf{L}_0}}\left[...E_{f_{\mathbf{L}_j,Y_j}}\left\{\sum_{a_j} Q_j \frac{\mathrm{d}q^g(a_j|\bar{\mathbf{L}}_j,\bar{A}_{j-1},\bar{Y}_j)}{\mathrm{d}t}\Big|_{t=0}|\bar{\mathbf{L}}_{j-1},\bar{A}_{j-1},\bar{Y}_{j-1}\right\}...\right]+ \tag{D.14}$$

$$\sum_{j=1}^{J-1} E_{f_{\mathbf{L}_0}}\left[...E_{f_{\mathbf{L}_j,Y_j}}\left\{E_{q^g_{A_j}}(Q_j|\bar{\mathbf{L}}_j,\bar{A}_{j-1},\bar{Y}_j)S_{\mathbf{L}_j,Y_j|\bar{\mathbf{L}}_{j-1},\bar{A}_{j-1},\bar{Y}_{j-1}}|\bar{\mathbf{L}}_{j-1},\bar{A}_{j-1},\bar{Y}_{j-1}\right\}...\right]+ \tag{D.15}$$

$$E_{f_{\mathbf{L}_0}}\left\{\sum_{a_0} Q_0 \frac{\mathrm{d}q^g(a_0|\mathbf{L}_0)}{\mathrm{d}t}\Big|_{t=0}\right\} + E_{f_{\mathbf{L}_0}}\left\{E_{q^g_{A_0}}(Q_0|\mathbf{L}_0)S_{\mathbf{L}_0}\right\} \tag{D.16}$$

Expression (D.13) can be shown to equal

$$\begin{aligned} &E\left[\frac{\prod_{j=0}^{J-1} q^g(A_j|Y_j=1,\bar{\mathbf{L}}_j,\bar{A}_{j-1})}{\prod_{j=0}^{J-1} f(A_j|Y_j=1,\bar{\mathbf{L}}_j,\bar{A}_{j-1})} E\left\{(Y_J - Q_{J-1})S_{Y_J|\bar{\mathbf{L}}_{J-1},\bar{A}_{J-1},\bar{Y}_{J-1}}|\bar{\mathbf{L}}_{J-1},\bar{A}_{J-1},\bar{Y}_{J-1}\right\}\right] \\ &= E\left\{\frac{\prod_{j=0}^{J-1} q^g(A_j|Y_j=1,\bar{\mathbf{L}}_j,\bar{A}_{j-1})}{\prod_{j=0}^{J-1} f(A_j|Y_j=1,\bar{\mathbf{L}}_j,\bar{A}_{j-1})}(Y_J - Q_{J-1})S_O\right\} \end{aligned} \tag{D.17}$$

Expression (D.14) can be shown to equal

$$\begin{aligned} &E\left[\frac{\prod_{k=0}^{j-1} q^g(A_k|Y_k=1,\bar{\mathbf{L}}_k,\bar{A}_{k-1})}{\prod_{j=0}^{j-1} f(A_k|Y_k=1,\bar{\mathbf{L}}_k,\bar{A}_{k-1})} E\left\{c_2 Q_j h_2(\bar{\mathbf{L}}_j,\bar{A}_{j-1})S_{A_j|\bar{\mathbf{L}}_j,\bar{A}_{j-1},\bar{Y}_j}|\bar{\mathbf{L}}_j,\bar{A}_{j-1},\bar{Y}_j\right\}\right] \\ &= E\left[\frac{\prod_{k=0}^{j-1} q^g(A_k|Y_k=1,\bar{\mathbf{L}}_k,\bar{A}_{k-1})}{\prod_{j=0}^{j-1} f(A_k|Y_k=1,\bar{\mathbf{L}}_k,\bar{A}_{k-1})} c_2\left\{Q_j - E(Q_j|\bar{\mathbf{L}}_j,\bar{A}_{j-1},\bar{Y}_j)\right\}h_2(\bar{\mathbf{L}}_j,\bar{A}_{j-1})S_O\right] \end{aligned}$$

Expression (D.15) can be shown to equal

$$E\left[\left\{\sum_{a_j} Q_j q^g(a_j|\bar{\mathbf{L}}_j,\bar{A}_{j-1},\bar{Y}_j{=}1) - Q_{j-1}\right\}\frac{\prod_{k=0}^{j-1} q^g(A_k|Y_k{=}1,\bar{\mathbf{L}}_k,\bar{A}_{k-1})}{\prod_{j=0}^{j-1} f(A_k|Y_k{=}1,\bar{\mathbf{L}}_k,\bar{A}_{k-1})} S_O\right]$$

$$= E\left[\left\{c_1 Q_j^{A_j=a_j^*} h_1(\bar{\mathbf{L}}_j,\bar{A}_{j-1}) + c_2 E(Q_j|\bar{\mathbf{L}}_j,\bar{A}_{j-1},\bar{Y}_j) h_2(\bar{\mathbf{L}}_j,\bar{A}_{j-1}) + \right.\right.$$

$$\left.\sum_{a_j} c_3 p^*(a_j|\bar{\mathbf{L}}_j,\bar{A}_{j-1},Y_j{=}1) Q_j^{A_j=a_j} h_3(\bar{\mathbf{L}}_j,\bar{A}_{j-1}) - Q_{j-1}\right\}$$

$$\left.\frac{\prod_{k=0}^{j-1} q^g(A_k|Y_k{=}1,\bar{\mathbf{L}}_k,\bar{A}_{k-1})}{\prod_{j=0}^{j-1} f(A_k|Y_k{=}1,\bar{\mathbf{L}}_k,\bar{A}_{k-1})} S_O\right]$$

Adding Expressions (D.14) and (D.15) together we get

$$E\left[\frac{\prod_{k=0}^{j-1} q^g(A_k|Y_k{=}1,\bar{\mathbf{L}}_k,\bar{A}_{k-1})}{\prod_{j=0}^{j-1} f(A_k|Y_k{=}1,\bar{\mathbf{L}}_k,\bar{A}_{k-1})}\left\{c_1 Q_j^{A_j=a_j^*} h_1(\bar{\mathbf{L}}_j,\bar{A}_{j-1}) + c_2 Q_j h_2(\bar{\mathbf{L}}_j,\bar{A}_{j-1}) + \right.\right.$$

$$\left.\left.\sum_{a_j} c_3 p^*(a_j|Y_j{=}1,\bar{\mathbf{L}}_j,\bar{A}_{j-1}) Q_j^{A_j=a_j} h_3(\bar{\mathbf{L}}_j,\bar{A}_{j-1}) - Q_{j-1}\right\} S_O\right] \tag{D.18}$$

In addition, it can be shown that (D.16) equals

$$E\left[\left\{c_1 Q_0^{A_0=a_0^*} h_1(L_0) + c_2 Q_0 h_2(\mathbf{L}_0) + \sum_{a_0} c_3 p^*(a_0|\mathbf{L}_0) Q_0^{A_0=a_0} h_3(\mathbf{L}_0)\right\} S_O\right] \tag{D.19}$$

By taking the sum of Expressions (D.17)–(D.19) over all j, it can be seen that the EIF of ψ^g is precisely given by Expression (9). □

E. Proof of model double robustness and model $J+1$ robustness of estimators based on the EIF for density under representation (8)

E.1 Proof of double robustness

Proof. Suppose that the estimates for Q_j, T_j, $f(A_j|Y_j{=}1,\bar{\mathbf{L}}_j,\bar{A}_{j-1})$ and $q^g(A_j|Y_j{=}1,\bar{\mathbf{L}}_j,\bar{A}_{j-1})$ are denoted by $\hat{Q}_j{=}E(\hat{T}_{j+1}|\bar{\mathbf{L}}_j,\bar{A}_j,\bar{Y}_j;\hat{\theta}_j)$, $\hat{T}_j$, $\pi_j(\hat{\alpha}_j)$ and $q^g_j(\hat{\alpha}_j)$, respectively. Let $\hat{\theta}{=}(\hat{\theta}_0,\ldots,\hat{\theta}_{J-1})$, $\hat{\alpha}{=}(\hat{\alpha}_0,\ldots,\hat{\alpha}_{J-1})$, and let $\psi^{g^\star}$, $\theta^\star{=}(\theta^\star_0,\ldots,\theta^\star_{J-1})$ and $\alpha^\star{=}(\alpha^\star_0,\ldots,\alpha^\star_{J-1})$ denote probability limits of $\hat{\psi^g}$, $\hat{\theta}$ and $\hat{\alpha}$, respectively. To first show that an estimator derived from and EIF is doubly robust, it suffices to show that following the estimating equations are unbiased when all of the treatment model is correctly specified and when all of the outcome regression models are correctly specified:

$$\mathbb{P}_n\left\{\underbrace{\sum_{j=1}^{J}(\hat{T}_j-\hat{Q}_{j-1})\frac{\prod_{k=0}^{j-1}q^g_k(\hat{\alpha}_k)}{\prod_{k=0}^{j-1}\pi_k(\hat{\alpha}_k)}}_{(\Delta)}+\hat{T}_0-\hat{\psi^g}\right\} \tag{E.20}$$

First, suppose that the outcome regression models are correctly specified at all time points such that $Q^\star_j{\equiv}E(T^\star_{j+1}|\bar{\mathbf{L}}_j,\bar{A}_j,\bar{Y}_j;\theta^\star){=}Q_j$ and $T^\star_j{=}T_j$ ($T^\star_j$ is a known function of $Q^\star_j$). It is easy to see that when the outcome regression models are correctly specified, for each $j{=}1,\ldots,J$,

$$\begin{aligned}&E\left\{(T^\star_j-Q^\star_{j-1})\frac{\prod_{k=0}^{j-1}q^g_k(\alpha^\star_k)}{\prod_{k=0}^{j-1}\pi_k(\alpha^\star_k)}|\bar{\mathbf{L}}_{j-1},\bar{A}_{j-1},Y_{j-1}\right\}\\&=E\left[\left\{E(T^\star_j|\bar{\mathbf{L}}_{j-1},\bar{A}_{j-1},Y_{j-1})-Q^\star_{j-1}\right\}\frac{\prod_{k=0}^{j-1}q^g_k(\alpha^\star_k)}{\prod_{k=0}^{j-1}\pi_k(\alpha^\star_k)}\right]=0\end{aligned}$$

Moreover, $E(T^\star_0-\psi^{g^\star}){=}0$ when $\psi^{g^\star}{=}\psi^g$ since $T^\star_0{=}T_0$.

Now consider that the treatment models are correctly specified at all time points such that $\pi_j(\alpha^\star_j){=}f(A_j|Y_j{=}1,\bar{\mathbf{L}}_j,\bar{A}_{j-1})$. Note that Equation (E.20) can be re-written as:

$$\mathbb{P}_n\left[\frac{\prod_{k=0}^{J-1}q^g_k(\hat{\alpha}_k)}{\prod_{k=0}^{j-1}\pi_k(\hat{\alpha}_k)}Y_J-\sum_{j=0}^{J-1}\frac{\prod_{k=0}^{j-1}q^g_k(\hat{\alpha}_k)}{\prod_{k=0}^{j-1}\pi_k(\hat{\alpha}_k)}\left\{\frac{q^g_k(\hat{\alpha}_j)}{\pi_k(\hat{\alpha}_j)}\hat{Q}_j-\hat{T}_j\right\}-\hat{\psi^g}\right]$$

For each $j{=}0,\ldots,J-1$, it can be shown that

$$\begin{aligned}&\frac{q^g_j(\alpha^\star_j)}{\pi_j(\alpha^\star_j)}Q^\star_j-T^\star_j\\&=c_1h_1(\bar{\mathbf{L}}_j,\bar{A}_{j-1})\left\{\frac{I_{\{a^*_j\}}(A_j)}{\pi_j(\alpha^\star_j)}-1\right\}Q^{\star A_j=a^*_j}_j+\sum_{a_j}c_{3,a_j}h_{3,a_j}(\bar{\mathbf{L}}_j,\bar{A}_{j-1})p^*(a_j|Y_j{=}1,\bar{\mathbf{L}}_j,\bar{A}_{j-1})\left\{\frac{I_{\{a_j\}}(A_j)}{\pi_j(\alpha^\star_j)}-1\right\}Q^{\star A_j=a_j}_j\end{aligned}$$

Taking the expectation of the last expression will yield zero under correct treatment models. Moreover, it can shown (just as for Horvitz-Thompson estimators) that under correctly specified treatment models,

$$E\left[\frac{\prod_{k=0}^{J-1}q^g_k(\alpha^\star_k)}{\prod_{k=0}^{j-1}\pi_k(\alpha^\star_k)}Y_J-\psi^{g^\star}\right]=0$$

when $\psi^{g^\star}{=}\psi^g$. □

E.2 Proof of $J{+}1$ multiple robustness

Proof. Let $\mathcal{H}_j$ correspond to a class of working models for propensity scores at time j that are correctly specified, and that $\mathcal{G}_j$ correspond to a class of working models for Q_j that are correctly specified. Suppose that outcome regression at the last j time points are correctly specified and that the propensity scores at the first $J{-}j$ time points are correctly specified (i.e. under $\{(\cap_{k=0}^{j-1}\mathcal{H}_k)\cap(\cap_{k=j}^{J-1}\mathcal{G}_k)\}$ for $j{=}0,\ldots,J$). For a follow-up of J time points, there are $2J{+}1$ terms in $\{\cdot\}$ in estimating equations (E.20). Taking conditional expectation at the limiting values of the parameters in the last three summands in (Δ), i.e.

$$E\left\{\prod_{k=0}^{J-1}\frac{q_k^g(\alpha_k^\star)}{\pi_k(\alpha_k^\star)}(Y_J-Q_{J-1}^\star)+\prod_{k=0}^{J-2}\frac{q_k^g(\alpha_k^\star)}{\pi_k(\alpha_k^\star)}T_{J-1}^\star \;\Big|\; \bar{A}_{J-1},\bar{Y}_{J-1},\bar{\mathbf{L}}_{J-1}\right\}$$

gives us:

$$\begin{aligned}&\prod_{k=0}^{J-2}\frac{q_k^g(\alpha_k^\star)}{\pi_k(\alpha_k^\star)}\left\{\frac{q_{J-1}^g(\alpha_{J-1}^\star)}{\pi_{J-1}(\alpha_{J-1}^\star)}\{E(Y_J|\bar{Y}_{J-1},\bar{A}_{J-1},\bar{\mathbf{L}}_{J-1})-Q_{J-1}^\star\}+T_{J-1}^\star\right\}\\&=\prod_{k=0}^{J-2}\frac{q_k^g(\alpha_k^\star)}{\pi_k(\alpha_k^\star)}T_{J-1}\end{aligned}$$

since $Q_{J-1}^\star{=}Q_{J-1}$ and $T_{J-1}^\star{=}T_{J-1}$ under $\mathcal{G}_{J-1}$. Using this result, taking conditional expectation of the last five summands in (Δ) of estimating equations (E.20) (given $\bar{A}_{J-2}$, $\bar{Y}_{J-2}$, and $\bar{\mathbf{L}}_{J-2}$) gives us:

$$\begin{aligned}&\prod_{k=0}^{J-3}\frac{q_k^g(\alpha_k^\star)}{\pi_k(\alpha_k^\star)}\left\{\frac{q_{J-2}^g(\alpha_{J-1}^\star)}{\pi_{J-2}(\alpha_{J-1}^\star)}\{E(T_{J-1}|\bar{Y}_{J-2},\bar{A}_{J-2},\bar{\mathbf{L}}_{J-2})-Q_{J-2}^\star\}+T_{J-2}^\star\right\}\\&=\prod_{k=0}^{J-3}\frac{q_k^g(\alpha_k^\star)}{\pi_k(\alpha_k^\star)}T_{J-2}\end{aligned}$$

under $\mathcal{G}_{J-1}\cap\mathcal{G}_{J-2}$. By induction, it can be shown that taking conditional expectation of the last $2(J{-}j){+}1$ terms in the summand (Δ) (given $\bar{A}_{j-1}$, $\bar{Y}_{j-1}$, and $\bar{\mathbf{L}}_{j-1}$) gives us $\prod_{k=0}^{j-1}\frac{q_k^g(\alpha_k^\star)}{\pi_k(\alpha_k^\star)}T_j$ under $\cap_{k=j}^{J-1}\mathcal{G}_k$.

Next, taking conditional expectation over the last $2(J{-}j){+}3$ summands in (Δ) of estimating equations (E.20) (given $\bar{A}_{j-2}$, $\bar{Y}_{j-2}$, and $\bar{\mathbf{L}}_{j-2}$) gives us:

$$\begin{aligned}&E\left[\prod_{k=0}^{j-2}\frac{q_k^g(\alpha_k^\star)}{\pi_k(\alpha_k^\star)}\left\{\frac{q_{j-1}^g(\alpha_{j-1}^\star)}{\pi_{j-1}(\alpha_{j-1}^\star)}(T_j-Q_{j-1}^\star)+T_{j-1}^\star\right\}\;\Big|\;\bar{A}_{j-2},\bar{Y}_{j-2},\bar{\mathbf{L}}_{j-2}\right]\\&=E\left[\prod_{k=0}^{j-2}\frac{q_k^g(\alpha_k^\star)}{\pi_k(\alpha_k^\star)}\left\{\frac{q_{j-1}^g(\alpha_{j-1}^\star)}{\pi_{j-1}(\alpha_{j-1}^\star)}T_j-\left(\frac{q_{j-1}^g(\alpha_{j-1}^\star)}{\pi_{j-1}(\alpha_{j-1}^\star)}Q_{j-1}^\star-T_{j-1}^\star\right)\right\}\;\Big|\;\bar{A}_{j-2},\bar{Y}_{j-2},\bar{\mathbf{L}}_{j-2}\right]\\&=\prod_{k=0}^{j-2}\frac{q_k^g(\alpha_k^\star)}{\pi_k(\alpha_k^\star)}E(T_{j-1}|\bar{A}_{j-2},\bar{Y}_{j-2},\bar{\mathbf{L}}_{j-2})=\prod_{k=0}^{j-2}\frac{q_k^g(\alpha_k^\star)}{\pi_k(\alpha_k^\star)}Q_{j-2}\end{aligned}$$

under $\mathcal{H}_{j-1}\cap(\cap_{k=j}^{J-1}\mathcal{G}_k)$. Finally, it can be shown that by induction, taking expectation over estimating equations (E.20) gives us

$$\begin{aligned}&E\left[\frac{q_0^g(\alpha_0^\star)}{\pi_0(\alpha_0^\star)}\left\{\frac{q_1^g(\alpha_1^\star)}{\pi_1(\alpha_1^\star)}Q_1-Q_0^\star\right\}+T_0^\star-\psi^{g^\star}\right]\\&=E(T_0)-\psi^{g^\star}\end{aligned}$$

which equals zero when $\psi^{g^\star}$ equals the true value ψ^g. □

E.3 Applying Theorem 2 to other intervention distributions

Example 1. *Consider the representative intervention given in Section 5 for* $J>1$*. The intervention distribution is given by* $q^g(a_j|1,\bar{\boldsymbol{l}}_j,\bar{a}_{j-1})=f(a_j|1,\bar{\boldsymbol{l}}_j,\bar{a}_{j-1},a_j\geq\delta)$

We note that the ψ^g under this intervention distribution takes the same form as the g-formula indexed by a static deterministic intervention that sets treatment to a fixed value but with with R_j playing the role of treatment at time j (see Young et al., 2019). Young *and others* showed that this implied intervention distribution is given by $\tilde{q}^g(r_j|1,\bar{\mathbf{l}}_j,\bar{r}_{j-1})=r_j$ where $R_j=I(A_j>\delta)$ and $\mathbf{L}_j$ contains A_{j-1}. Therefore, if we select $r_j^*=1$, $c_1=1$, $h_1(\bar{\boldsymbol{l}}_j,\bar{a}_{j-1})=1$, $h_2(\bar{\boldsymbol{l}}_j,\bar{r}_{j-1})=0$ and $h_3(\bar{\boldsymbol{l}}_j,\bar{r}_{j-1})=0$, then by Theorem 2, the EIF based on this implied intervention distribution is given by:

$$U_{\psi^g(\delta)}(O)=(Y_J-Q_{J-1})\prod_{j=0}^{J-1}\frac{R_j}{f(R_j|Y_j=1,\bar{\mathbf{L}}_j,\bar{R}_{j-1})}+\sum_{j=1}^{J-1}\Big(\underbrace{Q_j^{R_j=1}}_{T_j}-Q_{j-1}\Big)\prod_{k=0}^{j-1}\frac{R_k}{f(R_k|Y_k=1,\bar{\mathbf{L}}_k,\bar{R}_{k-1})}+\underbrace{Q_0^{R_0=1}}_{T_0}-\psi^g$$

Note that $Q_{j-1}\prod_{k=0}^{j-1}R_k=Q_{j-1}^{R_{j-1}=1}\prod_{k=0}^{j-1}R_k=T_j\prod_{k=0}^{j-1}R_k$, which means the above equation can be reduced to the following equation:

$$U_{\psi^g(\delta)}(O)=\sum_{j=1}^{J}(T_j-T_{j-1})\prod_{k=0}^{j-1}\frac{R_k}{f(R_k|Y_k=1,\bar{\mathbf{L}}_k,\bar{R}_{k-1})}+T_0-\psi^g$$

By Theorem 2, the estimators based on this EIF are multiply robust.

Example 2. *Consider the dynamic treatment initiation strategies described in Section 5 where treatment initiation within the grace period* m *follows the observed treatment process. The intervention distribution is given by* $q^g(a_j|1,\bar{\boldsymbol{l}}_j,\bar{a}_{j-1})=(1-l_j^*)(1-a_j)+l_{j-m}^*a_j+(1-l_{j-m}^*)l_j^*f(a_j|1,\bar{\boldsymbol{l}}_j,\bar{a}_{j-1})$.

Before deriving the EIF, we note that in Equation (8) if instead of $c_1I(a_j=a_j^*)h_1(\bar{\boldsymbol{l}}_j,\bar{a}_{j-1})$, we have $\sum_{m=1}^{v}c_{1,m}I(a_j=a_{j,m}^*)h_{1,m}(\bar{\boldsymbol{l}}_j,\bar{a}_{j-1})$ (that is, v deterministic rules assigned according to the intervention distribution), then we can replace $c_1Q_j^{A_j=a_j^*}h_1(\bar{\mathbf{L}}_j,\bar{A}_{j-1})$ in T_j with $\sum_{m=1}^{v}c_{1,m}Q_j^{A_j=a_{j,m}^*}h_{1,m}(\bar{\mathbf{L}}_j,\bar{A}_{j-1})$. The results from Theorem 2 still hold under this modification and can be trivially realized from the proof in D.

For instance, the intervention distribution for the aforementioned dynamic treatment initiation strategy with grace period can be written as Equation (8) but with $c_1I(a_j=a_j^*)h_1(\bar{\boldsymbol{l}}_j,\bar{a}_{j-1})$ replaced with $\sum_{m=1}^{2}c_{1,m}I(a_j=a_{j,m}^*)h_{1,m}(\bar{\boldsymbol{l}}_j,\bar{a}_{j-1})$ (i.e. $v=2$). This can be seen by selecting $a_{j,1}^*=0$, $a_{j,2}^*=1$, $c_{1,1}=c_{1,2}=1$, $h_{1,1}(\bar{\boldsymbol{l}}_j,\bar{a}_{j-1})=(1-l_j^*)$, $h_{1,2}(\bar{\boldsymbol{l}}_j,\bar{a}_{j-1})=l_{j-m}^*$, $h_2(\bar{\boldsymbol{l}}_j,\bar{a}_{j-1})=(1-l_{j-m}^*)l_j^*$ and $h_3(\bar{\boldsymbol{l}}_j,\bar{a}_{j-1})=0$. Then by Theorem 2, the EIF based on this intervention distribution is given by:

$$\begin{aligned}U_{\psi^g(\delta)}(O)=&(Y_J-Q_{J-1})\prod_{j=0}^{J-1}\frac{q^g(A_j|1,\bar{\mathbf{L}}_j,\bar{A}_{j-1})}{f(A_j|1,\bar{\mathbf{L}}_j,\bar{A}_{j-1})}+\\&\sum_{j=1}^{J-1}\Big\{\underbrace{Q_j^{A_j=0}(1-L_j^*)+Q_j^{A_j=1}L_{j-m}^*+Q_j(1-L_{j-m}^*)L_j^*}_{T_j}-Q_{j-1}\Big\}\prod_{k=0}^{j-1}\frac{q^g(A_k|1,\bar{\mathbf{L}}_k,\bar{A}_{k-1})}{f(A_k|1,\bar{\mathbf{L}}_k,\bar{A}_{k-1})}+\\&\underbrace{Q_0^{A_0=0}(1-L_0^*)+Q_0L_0^*}_{T_0}-\psi^g\end{aligned}$$

where it follows by definition that $\bar{L}_{-1}^*=0$ for all individuals (i.e. the threshold is not crossed prior to time 0). By Theorem 2, the estimators based on this EIF are multiply robust.

Example 3. *Deterministic dynamic treatment interventions*

Following the last example, any deterministic dynamic intervention distribution can be written as Equation (8) but with $c_1 I(a_j{=}a_j^*)h_1(\bar{\boldsymbol{l}}_j,\bar{a}_{j-1})$ replaced with $\sum_{m=1}^{v} c_{1,m} I(a_j{=}a_{j,m}^*)h_{1,m}(\bar{\boldsymbol{l}}_j,\bar{a}_{j-1})$. This can be seen by selecting $h_3(\bar{\boldsymbol{l}}_j,\bar{a}_{j-1}){=}h_2(\bar{\boldsymbol{l}}_j,\bar{a}_{j-1}){=}0$, $c_{1,1}{=}\ldots{,}{=}c_{1,v}{=}1$ and selecting $h_{1,m}(\bar{\boldsymbol{l}}_j,\bar{a}_{j-1})$ according to the intervention rule (for $m{=}1,\ldots,v$). For instance, if the deterministic dynamic treatment intervention is "treat when $L_j^*{=}1$ and do not treat when $L_j^*{=}0$", then the intervention distribution is given by $q^g(a_j| 1,\bar{\boldsymbol{l}}_j,\bar{a}_{j-1}){=}l_j^* a_j{+}(1{-}l_j^*)(1{-}a_j)$. If we select $v{=}2$, $a_{j,1}^*{=}1$, $a_{j,2}^*{=}0$, $h_{1,1}(\bar{l}_j,\bar{a}_{j-1}){=}l_j^*$ and $h_{1,2}(\bar{l}_j,\bar{a}_{j-1}){=}1{-}l_j^*$, then by Theorem 2, the EIF based on this intervention distribution is given by:

$$
\begin{aligned}
U_{\psi^g(\delta)}(O) = {} & (Y_J - Q_{J-1}) \prod_{j=0}^{J-1} \frac{q^g(A_j|1,\bar{\mathbf{L}}_j,\bar{A}_{j-1})}{f(A_j|1,\bar{\mathbf{L}}_j,\bar{A}_{j-1})} + \\
& \sum_{j=1}^{J-1} \Big\{ \underbrace{Q_j^{A_j=1} L_j^* + Q_j^{A_j=0}(1-L_j^*)}_{T_j} - Q_{j-1} \Big\} \prod_{k=0}^{j-1} \frac{q^g(A_k|1,\bar{\mathbf{L}}_k,\bar{A}_{k-1})}{f(A_k|1,\bar{\mathbf{L}}_k,\bar{A}_{k-1})} + \\
& \underbrace{Q_0 L_0^* + Q_0^{A_0=0}(1-L_0^*)}_{T_0} - \psi^g
\end{aligned}
$$

which can be shown to equal

$$
U_{\psi^g(\delta)}(O) = (Y_J - T_{J-1}) \frac{I(\bar{A}_{J-1}=\bar{L}_{J-1}^*)}{\prod_{j=0}^{J-1} f(A_j|1,\bar{\mathbf{L}}_j,\bar{A}_{j-1})} + \sum_{j=1}^{J-1} (T_j - T_{j-1}) \frac{I(\bar{A}_k=\bar{L}_k^*)}{\prod_{k=0}^{j-1} f(A_k|1,\bar{\mathbf{L}}_k,\bar{A}_{k-1})} + T_0 - \psi^g
$$

by realizing that $L_j^* A_j + (1-L_j^*)(1-A_j) = I(A_j = L_j^*)$.

F. Incorporating Machine Learning methods in the estimation procedure

In observational studies, misspecification in the functional form for the propensity score (π_j) and/or outcome regression models (Q_j) can induce biased estimates of the average treatment effect. In recent years, there has been an explosion in developing flexible data-adaptive methods (e.g. kernel smoothing, generalized additive models, ensemble learners, random forest) combined with doubly robust estimators that can reduce the risk of model misspecification and provide valid causal inference. These machine learning techniques offer more protection against model misspecficiation than the parametric models.

From first order expansion of a singly-robust plug-in estimator (IPW and ICE estimators), it can be shown that we require the nuisance parameter estimators to converge to the truth at rate $n^{-1/2}$. However, this is not possible for non-parametric conditional mean functions as this rate is not attainable for these types of functions. However when doubly robust estimators are used with data-adaptive methods this issue largely disappears are doubly robust estimators enjoy the small bias property (Newey et al., 2004).

In this section we will prove the asymptotic normality of our TMLE. Let

$$\varphi^g(\delta)=U_{\psi^g(\delta)}(O)+\psi^g(\delta),$$

We will prove the following Theorem.

Theorem 1. *Assume that the nuisance functions for π_j and Q_j are each consistently estimated at rates faster than $n^{-1/4}$ at each time point j ($j=0,\ldots,J-1$). Then*

$$\sqrt{n}\left\{\hat{\psi^g}_{TMLE}(\delta)-\psi^g(\delta)\right\}\rightsquigarrow N(0,\sigma^2_\delta)$$

where $\sigma^2_\delta=Var\{U_{\psi^g(\delta)}(O)\}$.

Note that the asymptotic variance can be estimated with $M^{-1}\sum_{m=1}^M\mathbb{P}_n^m\left[\{\hat{\varphi}^g_m(\delta)-\mathbb{P}_n^m(\hat{T}_0)\}^2\right]$, where $\hat{\varphi}^g_m(\delta)$ is the estimated value for $\varphi^g(\delta)$ in subsample m obtained from $\hat{\pi}_j^{(-m)}$, $\hat{Q}_j^{(-m)}$ and $\hat{\gamma}_j$ in the TMLE algorithm.

Proof. For notational brevity let $\hat{\psi^g}(\delta)\equiv\hat{\psi^g}_{TMLE}(\delta)$. Let $\hat{\Psi}^g_n(\delta)=\sqrt{n}\{\hat{\psi^g}(\delta)-\psi^g(\delta)\}$ and let $\Psi^g_n(\delta)=\sqrt{n}(\mathbb{P}_n-P)\{\varphi^g(\delta)-\psi^g(\delta)\}=\mathbb{G}_n[\varphi^g(\delta)-\psi^g(\delta)]$. To prove this Theorem, we will show that

$$\begin{aligned}\hat{\Psi}^g_n(\delta)&=\sqrt{n}\{\hat{\psi^g}(\delta)-\psi^g(\delta)\}\\&=\Psi^g_n(\delta)+\hat{\Psi}^g_n(\delta)-\Psi^g_n(\delta)\rightsquigarrow N(0,\sigma^2_\delta)\end{aligned}$$

To do this, we will first show that $\Psi^g_n(\delta)\rightsquigarrow N(0,\sigma^2_\delta)$ and then show that $\hat{\Psi}^g_n(\delta)-\Psi^g_n(\delta)=o_p(1)$. The result then follows from Slutsky's Theorem.

Step 1. $\Psi^g_n(\delta)\rightsquigarrow N(0,\sigma^2_\delta)$ follows from the Central Limit Theorem provided that $E\left\{U_{\psi^g(\delta)}(O)^2\right\}<\infty$. In addition, the class of functions $\mathcal{F}=\{\varphi^g(\delta):\delta\in[0,1]\}$ defined by δ is Donsker. This is true because $\mathcal{F}$ is also Lipschitz in δ (since the derivative of a function in this class taken with respect to δ can be shown to be bounded) and therefore by Example 19.7 in Van Der Vaart (2000), we know that the bracketing integral is finite: i.e. $J_{[]}(1,\mathcal{F},L_2(P))<\infty$, where $J_{[]}(1,\mathcal{F},L_2(P))=\int_0^1\sqrt{\log N_{[]}(\epsilon,\mathcal{F},L_2(P)}d\epsilon$. Therefore, by Theorem 19.5 of a Van Der Vaart (2000), the sequence

$$(\mathbb{G}_n[\varphi^g(\delta_1)-\psi^g(\delta_1)],\ldots,\mathbb{G}_n[\varphi^g(\delta_k)-\psi^g(\delta_k)])\rightsquigarrow\mathrm{GP}(0,K(\delta_s,\delta_t))$$

for $k\in\mathbb{N}$ where $\mathrm{GP}(0,K(\delta_s,\delta_t))$ is a mean zero Gaussian process with covariance given by $K(\delta_s,\delta_t)=E\{U_{\psi^g(\delta_s)}(O)U_{\psi^g(\delta_t)}(O)\}$. Therefore, for any value δ, we have asymptotic normality of $\Psi^g_n(\delta)$.

Step 2. By construction, we know that that $\mathbb{P}_n^m(\hat{T}_0) = \mathbb{P}_n^m\{\hat{\varphi}_m^g(\delta)\}$. Moreover, $\hat{\psi}^g(\delta) = M^{-1}\sum_{m=1}^M \mathbb{P}_n^m(\hat{T}_0) = M^{-1}\sum_{m=1}^M \mathbb{P}_n^m\{\hat{\varphi}_m^g(\delta)\}$. Then,

$$
\begin{aligned}
\hat{\Psi}_n^g(\delta) - \Psi_n^g(\delta) &= \sqrt{n}\Big\{\hat{\psi}^g(\delta) - \psi^g(\delta) - (\mathbb{P}_n - P)\varphi^g(\delta) + (\mathbb{P}_n - P)\psi^g(\delta)\Big\} \\
&= \sqrt{n}\Big\{\hat{\psi}^g(\delta) - \psi^g(\delta) - (\mathbb{P}_n - P)\varphi^g(\delta)\Big\} \\
&= \frac{\sqrt{n}}{M}\sum_{m=1}^M \left[\mathbb{P}_n^m\{\hat{\varphi}_m^g(\delta)\} - \psi^g(\delta) - \mathbb{P}_n\{\varphi^g(\delta)\} + P\{\varphi^g(\delta)\}\right] \\
&= \frac{\sqrt{n}}{M}\sum_{m=1}^M \left[\mathbb{P}_n^m\{\hat{\varphi}_m^g(\delta)\} - P\{\varphi^g(\delta)\} - \mathbb{P}_n^m\{\varphi^g(\delta)\} + P\{\varphi^g(\delta)\}\right] \\
&= \frac{\sqrt{n}}{M}\sum_{m=1}^M \left[\frac{1}{\sqrt{|S_m|}}\mathbb{G}_n^m\{\hat{\varphi}_m^g(\delta) - \varphi^g(\delta)\} + P\{\hat{\varphi}_m^g(\delta) - \varphi^g(\delta)\}\right] \\
&= R_{1n} + R_{2n}
\end{aligned}
$$

where $\mathbb{G}_n^m(\cdot) = \sqrt{|S_m|}(\mathbb{P}_n^m - P)(\cdot)$.

If an estimator for $\varphi^g(\delta)$ belongs to Donsker classes, then sample splitting is not required and by Lemma 19.24 in Van Der Vaart (2000) $R_{1n} = o_p(1)$. However, overfitting the estimators $(\hat{\pi}_j, \hat{Q}_j)$ for the nuisance functions from machine learning algorithms can lead to bias. This bias arise because the same data are used to estimate $\varphi^g(\delta)$ and $(\hat{\pi}_j, \hat{Q}_j)$. To remove the bias due to overfitting, we employ sample splitting and cross-fitting (Chernozhukov et al., 2018), thereby making the data in S_m independent of $\hat{\pi}_j^{(-m)}$ and $\hat{Q}_j^{(-m)}$. We change notations slightly to show $R_{1n} = o_p(1)$. In particular, let $\hat{\varphi}_m^g(\delta) = \varphi_m^g(\hat{\eta}^{(-m)}, \hat{\gamma}; \delta)$ where $\hat{\eta}^{(-m)}$ are the vector of estimated nuisance functions obtained from the training sample. Then by Z-estimation theory, we know that $\sqrt{|S_m|}(\hat{\gamma}_j - \gamma_j) = O_p(1)$ where $\gamma_j = 0$ for all j. It follows from the law of large numbers and the continuous mapping theorem that $\mathbb{G}_n^m\Big\{\varphi_m^g(\hat{\eta}^{(-m)}, \hat{\gamma}; \delta) - \varphi^g(\delta)\Big\} = \mathbb{G}_n^m\Big\{\varphi_m^g(\hat{\eta}^{(-m)}, \gamma; \delta) - \varphi^g(\delta)\Big\} + o_p(1)$. The rest of the proof to show $R_{1n} = o_p(1)$ follows the line of reasoning in proof of Theorem 2.1 (and Lemma B.1) in Chernozhukov et al. (2017). Conditioning on the data frome training subset S_{-m}, it can be shown that conditional expectation of the $\mathbb{G}_n^m\Big\{\varphi_m^g(\hat{\eta}^{(-m)}, \gamma; \delta) - \varphi^g(\delta)\Big\}$ is zero and its conditional variance goes to 0 in probability provided that $E[\{\varphi_m^g(\hat{\eta}^{(-m)}, \gamma; \delta) - \varphi(\delta)\}^2] \xrightarrow{p} 0$. An application of Chebyshev's inequality then shows that $R_{1n} = o_p(1)$.

Next we show that $R_{2n} = o_p(1)$ under some conditions about the convergence rates of the nuisance functions. First we will modify some notations for simplicity. Let $\psi^g(\delta) = \psi(P)$, $U_{\psi^g}(\delta)(O) = \psi_P^1$, $\hat{\psi}^g(\delta) = \psi(\hat{P})$ and $U_{\hat{\psi}^g}(\delta)(O) = \psi_{\hat{P}}^1$. By definition,

$$
P\{\hat{\varphi}^g(\delta) - \varphi^g(\delta)\} = E(\psi_{\hat{P}}^1) + \psi(\hat{P}) - \psi(P)
$$

where the expectation (as with the ones below) are taken with respect to the true data generating law P. For the proposed incremental propensity score intervention,

$$
\psi_P^1 = \sum_{j=1}^J \left(T_j - Q_{j-1}\right)\prod_{k=0}^{j-1}\frac{q_k^g}{\pi_k} + T_0 - \psi^g
$$

where

$$
T_J = Y_J,\ T_j = Q_j(L_j^*\delta + 1 - L_j^*) + Q_j^{A_j=1}L_j^*(1-\delta)\ \text{,for } j \le J-1,
$$

$$
q_j^g = q^g(A_j | Y_j = 1, \bar{\mathbf{L}}_j, \bar{A}_{j-1}),\ \pi_j = f(A_j | Y_j = 1, \bar{\mathbf{L}}_j, \bar{A}_{j-1})\ \text{,for } j \le J-1.
$$

Note that $\psi^g = E(T_0)$. Therefore,

$$E(\psi^1_{\hat{P}}) + \psi(\hat{P}) - \psi(P) = E\left\{\sum_{j=1}^{J}\left(\hat{T}_j - \hat{Q}_{j-1}\right)\prod_{k=0}^{j-1}\frac{\hat{q}^g_k}{\hat{\pi}_k} + \hat{T}_0 - T_0\right\}$$

$$= E\left\{\left(\hat{T}_1 - T_1 + T_1 - Q_0 + Q_0 - \hat{Q}_0\right)\frac{\hat{q}^g_0}{\pi_0} + \hat{T}_0 - T_0\right\} + \underbrace{E\left\{\sum_{j=2}^{J}\left(\hat{T}_j - \hat{Q}_{j-1}\right)\prod_{k=0}^{j-1}\frac{\hat{q}^g_k}{\hat{\pi}_k}\right\}}_{①}$$

$$= E\left\{\left(\hat{T}_1 - T_1 + Q_0 - \hat{Q}_0\right)\left\{L^*_0\delta + 1 - L^*_0 + (1-\delta)L^*_0\frac{A_0}{\hat{\pi}_0}\right\} + \hat{T}_0 - T_0 + ①\right\}$$

$$= E\Bigg\{\left(\hat{T}_1 - T_1\right)\left\{L^*_0\delta + 1 - L^*_0 + (1-\delta)L^*_0\frac{A_0}{\hat{\pi}_0}\right\} + \left(Q_0 - \hat{Q}_0\right)(L^*_0\delta + 1 - L^*_0) +$$

$$\left(Q_0^{A_0=1} - \hat{Q}_0^{A_0=1}\right)\left\{(1-\delta)L^*_0\frac{A_0}{\hat{\pi}_0}\right\} + \left(\hat{Q}_0 - Q_0\right)(L^*_0\delta + 1 - L^*_0) -$$

$$\left(\hat{Q}_0^{A_0=1} - Q_0^{A_0=1}\right)(1-\delta)L^*_0\Bigg\} + ①$$

$$= E\left\{\left(\hat{T}_1 - T_1\right)\frac{\hat{q}^g_0}{\pi_0}\right\} + \underbrace{E\left\{(1-\delta)L^*_0\left(Q_0^{A_0=1} - \hat{Q}_0^{A_0=1}\right)\left(\frac{A_0}{\hat{\pi}_0} - 1\right)\right\}}_{\Delta_1} + ①$$

$$= \Delta_1 + E\left\{\left(\hat{T}_1 - T_1\right)\frac{\hat{q}^g_0}{\pi_0}\right\} + E\left\{(\hat{T}_2 - T_2 + T_2 - Q_1 + Q_1 - \hat{Q}_1)\prod_{k=0}^{1}\frac{\hat{q}^g_k}{\hat{\pi}_k}\right\}$$

$$\underbrace{E\left\{\sum_{j=3}^{J}\left(\hat{T}_j - \hat{Q}_{j-1}\right)\prod_{k=0}^{j-1}\frac{\hat{q}^g_k}{\hat{\pi}_k}\right\}}_{②}$$

$$= \Delta_1 + ② + E\left\{(\hat{T}_2 - T_2)\prod_{k=0}^{1}\frac{\hat{q}^g_k}{\hat{\pi}_k}\right\} +$$

$$E\Bigg[\frac{\hat{q}^g_0}{\hat{\pi}_0}(Q_1 - \hat{Q}_1)\left\{(L^*_1\delta + 1 - L^*_1) + (1-\delta)L^*_1\frac{A_1}{\hat{\pi}_1}(Q_1^{A_1=1} - \hat{Q}_1^{A_1=1})\right\} +$$

$$\frac{\hat{q}^g_0}{\hat{\pi}_0}\left\{(\hat{Q}_1 - Q_1)(L^*_1\delta + 1 - L^*_1) + (1-\delta)L^*_1(\hat{Q}_1^{A_1=1} - Q_1^{A_1=1})\right\}\Bigg]$$

$$= \Delta_1 + ② + E\left\{(\hat{T}_2 - T_2)\prod_{k=0}^{1}\frac{\hat{q}^g_k}{\hat{\pi}_k}\right\} + E\left\{\frac{\hat{q}^g_0}{\hat{\pi}_0}(1-\delta)L^*_1(\hat{Q}_1^{A_1=1} - Q_1^{A_1=1})\left(\frac{A_1}{\hat{\pi}_1} - 1\right)\right\}$$

Induction hypothesis: for any $r < J$, suppose that $E(\psi^1_{\hat{P}}) + \psi(\hat{P}) - \psi(P)$ can be written as:

$$\underbrace{\sum_{j=0}^{r-1}E\left\{\prod_{k=0}^{j-1}\frac{\hat{q}^g_k}{\hat{\pi}_k}(1-\delta)L^*_j(\hat{Q}_j^{A_j=1} - Q_j^{A_j=1})\left(\frac{A_j}{\hat{\pi}_j} - 1\right)\right\}}_{\Delta_2} + E\left\{(\hat{T}_r - T_r)\prod_{k=0}^{r-1}\frac{\hat{q}^g_k}{\hat{\pi}_k}\right\} + E\left\{\sum_{j=r+1}^{J}\left(\hat{T}_j - \hat{Q}_{j-1}\right)\prod_{k=0}^{j-1}\frac{\hat{q}^g_k}{\hat{\pi}_k}\right\}$$

We shall show that this previous expression also holds for $r+1$. From the induction hypothesis, $E(\psi^1_{\hat{P}})+\psi(\hat{P})-\psi(P)$ then equals

$$\Delta_2+E\left\{(\hat{T}_r-T_r)\prod_{k=0}^{r-1}\frac{\hat{q}_k^g}{\hat{\pi}_k}\right\}+E\left\{(\hat{T}_{r+1}-T_{r+1}+T_{r+1}-Q_r+Q_r-\hat{Q}_r)\prod_{k=0}^{r}\frac{\hat{q}_k^g}{\hat{\pi}_k}\right\}+\underbrace{E\left\{\sum_{j=r+2}^{J}\left(\hat{T}_j-\hat{Q}_{j-1}\right)\prod_{k=0}^{j-1}\frac{\hat{q}_k^g}{\hat{\pi}_k}\right\}}_{③}$$

$$=\Delta_2+③+E\left\{(\hat{T}_{r+1}-T_{r+1})\prod_{k=0}^{r}\frac{\hat{q}_k^g}{\hat{\pi}_k}\right\}+$$

$$E\left[\prod_{k=0}^{r-1}\frac{\hat{q}_k^g}{\hat{\pi}_k}(Q_r-\hat{Q}_r)\left\{(L_r^*\delta+1-L_r^*)+(1-\delta)L_r^*\frac{A_r}{\hat{\pi}_r}(Q_r^{A_r=1}-\hat{Q}_r^{A_r=1})\right\}+\right.$$

$$\left.\prod_{k=0}^{r-1}\frac{\hat{q}_k^g}{\hat{\pi}_k}\left\{(\hat{Q}_r-Q_r)(L_r^*\delta+1-L_r^*)+(1-\delta)L_r^*(\hat{Q}_r^{A_r=1}-Q_r^{A_r=1})\right\}\right]$$

$$=\sum_{j=0}^{r}E\left\{\prod_{k=0}^{j-1}\frac{\hat{q}_k^g}{\hat{\pi}_k}(1-\delta)L_j^*(\hat{Q}_j^{A_j=1}-Q_j^{A_j=1})\left(\frac{A_j}{\hat{\pi}_j}-1\right)\right\}+E\left\{(\hat{T}_{r+1}-T_{r+1})\prod_{k=0}^{r}\frac{\hat{q}_k^g}{\hat{\pi}_k}\right\}+E\left\{\sum_{j=r+2}^{J}\left(\hat{T}_j-\hat{Q}_{j-1}\right)\prod_{k=0}^{j-1}\frac{\hat{q}_k^g}{\hat{\pi}_k}\right\}$$

Therefore,

$$E(\psi^1_{\hat{P}})+\psi(\hat{P})-\psi(P)=\sum_{j=0}^{J-1}E\left\{\prod_{k=0}^{j-1}\frac{\hat{q}_k^g}{\hat{\pi}_k}(1-\delta)L_j^*(\hat{Q}_j^{A_j=1}-Q_j^{A_j=1})\left(\frac{A_j}{\hat{\pi}_j}-1\right)\right\}$$

Let $\pi_{j,1}=P(A_j=1|\bar{A}_{j-1},\bar{L}_Y,Y_j=1)$ and similarly let $\hat{\pi}_{j,1}=\hat{P}(A_j=1|\bar{A}_{j-1},\bar{L}_Y,Y_j=1)$. For each jth term,

$$\begin{aligned}E\left\{\prod_{k=0}^{j-1}\frac{\hat{q}_k^g}{\hat{\pi}_k}(1-\delta)L_j^*(\hat{Q}_j^{A_j=1}-Q_j^{A_j=1})\left(\frac{A_j}{\hat{\pi}_j}-1\right)\right\}&=E\left\{\prod_{k=0}^{j-1}\frac{\hat{q}_k^g}{\hat{\pi}_k}(1-\delta)A_jL_j^*(\hat{Q}_j^{A_j=1}-Q_j^{A_j=1})\left(\frac{1}{\hat{\pi}_{j,1}}-\frac{1}{\pi_{j,1}}\right)\right\}\\&=E\left\{\prod_{k=0}^{j-1}\frac{\hat{q}_k^g}{\hat{\pi}_k}(1-\delta)A_jL_j^*(\hat{Q}_j^{A_j=1}-Q_j^{A_j=1})\left(\hat{\pi}_{j,1}-\pi_{j,1}\right)\frac{1}{\hat{\pi}_{j,1}\pi_{j,1}}\right\}\\&\lesssim\left\|\hat{Q}_j^{A_j=1}-Q_j^{A_j=1}\right\|\,\left\|\hat{\pi}_{j,1}-\pi_{j,1}\right\|\end{aligned}$$

where $\|f(x)\|=\{\int|f(x)|^2dP(x)\}^{1/2}$, i.e. the $L_2(P)$ norm, and the last inequality (bounded above up to a constant) holds by an application of Cauchy-Schwartz. Therefore, for all values of j, as long as

$$\left\|\hat{Q}_j^{A_j=1}-Q_j^{A_j=1}\right\|\,\left\|\hat{\pi}_{j,1}-\pi_{j,1}\right\|=O_p(n^{-\nu}),\text{ for }\nu>1/2,$$

then $\sqrt{n}\{E(\psi^1_{\hat{P}})+\psi(\hat{P})-\psi(P)\}=o_p(1)$. This can be accomplished, for example, if the nuisance functions are each consistently estimated at a rate of $n^{-1/4}$ or faster. When there is censoring, the expansion of $E(\psi^1_{\hat{P}})+\psi(\hat{P})-\psi(P)$ is slightly different. In this case it is required that

$$\max\left\{\left\|\hat{Q}_j^{A_j=1}-Q_j^{A_j=1}\right\|\,\left\|\hat{\pi}_{j,1}\hat{\pi}_j^c-\pi_{j,1}\pi_j^c\right\|,\,\left\|\hat{Q}_j-Q_j\right\|\,\left\|\hat{\pi}_j^c-\pi_j^c\right\|\right\}=O_p(n^{-\nu}),\text{ for }\nu>1/2.$$

□

G. Modification to allow for censoring

We implicitly assume that all strategies include a hypothetical intervention that removes censoring through follow-up. In addition to estimating α_j in $f(A_j|\bar{A}_{j-1},\bar{\mathbf{L}}_j,C_j{=}0,Y_j{=}1;\alpha_j)$, we will also need to estimate α^c_j in $\pi^c_j(\alpha^c{}_j)\equiv P(C_{j+1}{=}1|C_j{=}0,\bar{A}_j,\bar{\mathbf{L}}_j,Y_j{=}1;\alpha^c_j)$ for $\pi^c_j\equiv P(C_{j+1}{=}1|C_j{=}0,\bar{A}_j,\bar{\mathbf{L}}_j,Y_j{=}1)$ $(j{=}0,\ldots,J{-}1)$. Let $\alpha^c{=}(\alpha^c_1,\ldots,\alpha^c_J)$. We modify the algorithm to be the following

Algorithm 1: Algorithm for Weighted ICE with censoring

1. Compute the MLEs $(\hat{\alpha},\hat{\alpha^c})$ of (α,α^c) from the observed data. Set $\hat{T}_J{=}Y_J$ and $q{=}1$.
2. Recursively from $j{=}J{-}1,\ldots,0$:
   a). Fit a logistic regression model $Q_j(\bar{\mathbf{L}}_j,\bar{A}_j,C_{j+1}{=}0,Y_j{=}1;\theta_j){=}\text{expit}\{\theta_j^T\phi(\bar{\mathbf{L}}_j,\bar{A}_j)\}$ for $E(\hat{T}_{j+1}|\bar{\mathbf{L}}_j,\bar{A}_j,C_{j+1}{=}0,Y_j{=}1)$ with observational weight $(1{-}C_{j+1})\prod_{k=0}^j\hat{q}^g_k/\prod_{k=0}^j\hat{\pi}_k\hat{\pi}^c_k$ in those who survive by time j. Here, $\phi(\bar{\mathbf{L}}_j,\bar{A}_j)$ is a known function of $\bar{\mathbf{L}}_j$ and $\bar{A}_j$. More specifically, we solve for θ_j in the following estimating equation using a weighted regression model:
   $$\mathbb{P}_n\left[Y_j\frac{\prod_{k=0}^j\hat{q}^g}{\prod_{k=0}^j\hat{\pi}_k\hat{\pi}^c_k}\phi_j(\bar{\mathbf{L}}_j,\bar{A}_j)\left\{\hat{T}_{j+1}-Q_j(\bar{\mathbf{L}}_j,\bar{A}_j;\theta_j)\right\}\right]=0$$
   b). Predict $\hat{T}_j$ from $\hat{Q}_j\equiv Q_j(\bar{\mathbf{L}}_j,\bar{A}_j,C_{j+1}{=}0,\bar{Y}_j;\hat{\theta}_j)$ as defined by the intervention in those uncensored at time j. Set $\hat{T}_j{=}0$ if $Y_j{=}0$.
3. Estimate $\hat{\psi}^g(\delta){=}\mathbb{P}_n(\hat{T}_0)$

To accommodate machine learning algorithms, the TMLE can be modified to be the following:

Algorithm 2: Algorithm for TMLE with censoring

1. For each $m{=}1,\ldots,M$:
   a). ***For individuals in*** S_{-m}: predict $\hat{\pi}_j^{(-m)}$ and $\hat{\pi}_j^{c(-m)}$, $\forall j$. Set $\hat{T}_J{=}Y_J$.
   b). Recursively from $j{=}J{-}1,\ldots,0$ ***for individuals in*** S_{-m}:
      (i) Compute $\hat{Q}_j^{(-m)}(\bar{\mathbf{L}}_j,\bar{A}_j,C_{j+1}{=}0,Y_j{=}1)$ for $E(\hat{T}_{j+1}|\bar{\mathbf{L}}_j,\bar{A}_j,C_{j+1}{=}0,Y_j{=}1)$ in those alive at time j and uncensored at time $j{+}1$
      (ii) Predict $\hat{T}_j$ using $\hat{Q}_j^{(-m)}\equiv\hat{Q}_j^{(-m)}(\bar{\mathbf{L}}_j,\bar{A}_j,\bar{C}_{j+1},\bar{Y}_j)$ in those who remain uncensored at time j, setting $\hat{T}_j{=}0$ if $Y_j{=}0$.
   c). ***For individuals in*** S_m, set $\hat{T}_J{=}Y_j$, and recursively from $j{=}J{-}1,\ldots,0$:
      (i) Solve for γ_j in the following set of estimating equations:
$$\mathbb{P}_n^m\left(Y_j\frac{(1{-}C_{j+1})\prod_{k=0}^j\hat{q}_k^{g(-m)}}{\prod_{k=0}^j\hat{\pi}_k^{(-m)}\hat{\pi}_k^{c(-m)}}\left[\hat{T}_{j+1}-\text{expit}\left\{\text{logit}\left(\hat{Q}_j^{(-m)}(\bar{\mathbf{L}}_j,\bar{A}_j,C_{j+1}{=}0,Y_j{=}1)\right)+\gamma_j\right\}\right]\right)=0$$
      (ii) Predict $\hat{T}_j$ from
      $\hat{Q}_j^{\Delta}(\bar{\mathbf{L}}_j,\bar{A}_j,C_{j+1}{=}0,Y_j{=}1)\equiv\text{expit}\left\{\text{logit}\left(\hat{Q}_j^{(-m)}(\bar{\mathbf{L}}_j,\bar{A}_j,C_{j+1}{=}0,Y_j{=}1)\right)+\hat{\gamma}_j\right\}$ in those uncensored at time and alive at time j, and set $\hat{T}_j{=}0$ if $C_j{=}Y_j{=}0$
2. Calculate
$$\hat{\psi}^g(\delta)_{TMLE}=\frac{1}{M}\sum_{m=1}^M\mathbb{P}_n^m(\hat{T}_0)$$

H. Results from simulation study

Tables 3–5 show the results from the simulation settings described in the main text where we considered scenarios where 1) all models are correctly specified, 2) only treatment models are correctly specified and 3) only outcome regression models are correctly specified for n=500, 1000, 2500. In Table 6, we consider an extra scenario where the outcome regression models are correctly specified at time 2 and after, and treatment models are correctly specified before time 2.

Table 3. Simulation study for proposed intervention (n=500). True 5-year probability of survival is (0.208, 0.263, 0.326) for δ=(0.75, 0.50, 0.25). All values are multiplied by 100.

	All correct				Treatment misspec.				Outcome misspec.			
δ=0.75												
Estimator	BIAS	SE	RMSE	MAE	BIAS	SE	RMSE	MAE	BIAS	SE	RMSE	MAE
$\hat{\psi}^g(\delta)_{ICE}$	0.00	2.15	2.15	1.73	0.00	2.15	2.15	1.73	-1.47	1.97	2.46	2.00
$\hat{\psi}^g(\delta)_{IPW}$	-0.11	2.75	2.76	2.17	-0.70	2.66	2.75	2.17	-0.11	2.75	2.76	2.17
$\hat{\psi}^g(\delta)_{WICE}$	-0.06	2.59	2.59	2.06	-0.05	2.50	2.51	1.99	-0.15	2.60	2.60	2.06
δ=0.50												
$\hat{\psi}^g(\delta)_{ICE}$	-0.06	2.75	2.75	2.21	-0.06	2.75	2.75	2.21	-3.31	2.27	4.01	3.46
$\hat{\psi}^g(\delta)_{IPW}$	-0.27	4.21	4.22	3.32	-1.28	4.45	4.63	3.65	-0.27	4.21	4.22	3.32
$\hat{\psi}^g(\delta)_{WICE}$	-0.19	3.77	3.77	2.99	-0.17	3.70	3.70	2.96	-0.34	3.86	3.88	3.07
δ=0.25												
$\hat{\psi}^g(\delta)_{ICE}$	-0.16	3.60	3.61	2.87	-0.16	3.60	3.61	2.87	-5.50	2.68	6.12	5.55
$\hat{\psi}^g(\delta)_{IPW}$	-0.39	6.15	6.16	4.85	-2.16	7.04	7.36	5.75	-0.39	6.15	6.16	4.85
$\hat{\psi}^g(\delta)_{WICE}$	-0.44	5.34	5.36	4.24	-0.38	5.37	5.38	4.30	-0.54	5.55	5.58	4.41

Table 4. Simulation study for proposed intervention (n=1000). True 5-year probability of survival is (0.208, 0.263, 0.326) for δ=(0.75, 0.50, 0.25). All values are multiplied by 100.

	All correct				Treatment misspec.				Outcome misspec.			
δ=0.75												
Estimator	BIAS	SE	RMSE	MAE	BIAS	SE	RMSE	MAE	BIAS	SE	RMSE	MAE
$\hat{\psi}^g(\delta)_{ICE}$	-0.07	1.47	1.47	1.18	-0.07	1.47	1.47	1.18	-1.52	1.35	2.04	1.70
$\hat{\psi}^g(\delta)_{IPW}$	-0.10	1.94	1.94	1.57	-0.72	1.85	1.98	1.59	-0.10	1.94	1.94	1.57
$\hat{\psi}^g(\delta)_{WICE}$	-0.09	1.83	1.83	1.48	-0.10	1.72	1.72	1.39	-0.12	1.84	1.84	1.49
δ=0.50												
$\hat{\psi}^g(\delta)_{ICE}$	-0.12	1.91	1.91	1.54	-0.12	1.91	1.91	1.54	-3.35	1.57	3.70	3.37
$\hat{\psi}^g(\delta)_{IPW}$	-0.20	3.08	3.08	2.46	-1.27	3.23	3.47	2.76	-0.20	3.08	3.08	2.46
$\hat{\psi}^g(\delta)_{WICE}$	-0.21	2.78	2.79	2.24	-0.21	2.61	2.62	2.09	-0.24	2.84	2.85	2.30
δ=0.25												
$\hat{\psi}^g(\delta)_{ICE}$	-0.07	1.47	1.47	1.18	-0.07	1.47	1.47	1.18	-5.53	1.88	5.84	5.53
$\hat{\psi}^g(\delta)_{IPW}$	-0.28	4.64	4.64	3.69	-2.16	5.32	5.75	4.48	-0.28	4.64	4.64	3.69
$\hat{\psi}^g(\delta)_{WICE}$	-0.38	4.05	4.07	3.28	-0.34	3.91	3.93	3.15	-0.37	4.17	4.18	3.38

Table 5. Simulation study for proposed intervention (n=2500). True 5-year probability of survival is (0.208, 0.263, 0.326) for δ=(0.75, 0.50, 0.25). All values are multiplied by 100.

	All correct				Treatment misspec.				Outcome misspec.			
δ=0.75												
Estimator	BIAS	SE	RMSE	MAE	BIAS	SE	RMSE	MAE	BIAS	SE	RMSE	MAE
$\hat{\psi}^g(\delta)_{ICE}$	−0.02	0.97	0.97	0.76	−0.02	0.97	0.97	0.76	−1.48	0.91	1.74	1.53
$\hat{\psi}^g(\delta)_{IPW}$	−0.05	1.24	1.24	0.97	−0.67	1.17	1.35	1.08	−0.05	1.24	1.24	0.97
$\hat{\psi}^g(\delta)_{WICE}$	−0.04	1.18	1.18	0.93	−0.05	1.09	1.10	0.87	−0.05	1.18	1.18	0.93
δ=0.50												
$\hat{\psi}^g(\delta)_{ICE}$	−0.05	1.23	1.24	0.98	−0.05	1.23	1.24	0.98	−3.30	1.05	3.47	3.30
$\hat{\psi}^g(\delta)_{IPW}$	−0.12	1.98	1.99	1.57	−1.19	2.13	2.44	1.91	−0.12	1.98	1.99	1.57
$\hat{\psi}^g(\delta)_{WICE}$	−0.10	1.84	1.84	1.46	−0.10	1.73	1.73	1.38	−0.11	1.85	1.86	1.47
δ=0.25												
$\hat{\psi}^g(\delta)_{ICE}$	−0.06	1.61	1.61	1.29	−0.06	1.61	1.61	1.29	−5.48	1.25	5.62	5.48
$\hat{\psi}^g(\delta)_{IPW}$	−0.17	3.07	3.07	2.41	−2.10	3.79	4.33	3.32	−0.17	3.07	3.07	2.41
$\hat{\psi}^g(\delta)_{WICE}$	−0.17	2.76	2.76	2.19	−0.15	2.71	2.71	2.17	−0.17	2.80	2.81	2.23

Table 6. Simulation study for proposed intervention (n=2500) for extra scenario. True 5-year probability of survival is (0.208, 0.263, 0.326) for δ=(0.75, 0.50, 0.25). All values are multiplied by 100.

	δ=0.75				δ=0.5				δ=0.25			
Estimator	BIAS	SE	RMSE	MAE	BIAS	SE	RMSE	MAE	BIAS	SE	RMSE	MAE
$\hat{\psi}^g(\delta)_{ICE}$	-1.13	0.92	1.46	1.24	-2.53	1.10	2.76	2.54	-4.19	1.35	4.40	4.19
$\hat{\psi}^g(\delta)_{IPW}$	-0.48	1.28	1.37	1.09	-0.87	2.43	2.58	2.01	-1.68	4.26	4.57	3.48
$\hat{\psi}^g(\delta)_{WICE}$	-0.07	1.17	1.18	0.93	-0.14	1.92	1.92	1.54	-0.20	2.98	2.99	2.38

I. Additional results from data application

Table 7. Relative survival estimates (compared with δ=1) and 95% confidence intervals from analysis of MSM from the Cambridge Health Alliance on the effect of incremental PrEP initiation on incident STI diagnosis.

	$\hat{\psi}^g(\delta)_{TMLE}$ (with ML)		$\hat{\psi}^g(\delta)_{WICE}$		$\hat{\psi}^g(\delta)_{ICE}$		$\hat{\psi}^g(\delta)_{IPW}$	
↑ in PrEP	Est.	95% C.I.	Est.	95% C.I.	Est.	95% C.I.	Est.	95% C.I.
Low	0.99	(0.96, 1.01)	0.99	(0.98, 1.00)	0.99	(0.98, 1.00)	0.99	(0.97, 1.00)
Medium	0.97	(0.91, 1.01)	0.98	(0.93, 1.01)	0.98	(0.95, 1.00)	0.97	(0.92, 1.01)
High	0.96	(0.87, 1.02)	0.96	(0.89, 1.01)	0.96	(0.92, 1.00)	0.96	(0.87, 1.01)

I.1 Summary of weights from $\prod_{k=0}^{j}(\hat{q}_k^g/\hat{\pi}_k)$, for j=0,...,$J-1$ in those whose L_j^*=1.

Here, we show a summary of estimated inverse probability weights from the IPW estimator for all time points:

Table 8. Summary of inverse probability weights for δ=(0.95, 0.85, 0.75). Values in brackets are the ranges that are observed across all time point.

δ	Min.	1st Quant.	Median	3rd Quant.	Max
0.95	(0.74–0.95)	(0.84–0.89)	(0.90–1.78)	(0.90–7.00)	(0.90–20.20)
0.85	(0.38–0.85)	(0.57–0.85)	(0.72–3.07)	(0.72–15.74)	(0.72–47.07)
0.75	(0.18–0.75)	(0.37–0.75)	(0.56–4.02)	(0.56–20.64)	(0.56–60.70)

References

Cain L, Robins JM, Lanoy E, Logan R, Costagliola D and Hernán MA (2010) When to start treatment? a systematic approach to the comparison of dynamic regimes using observational data. *The International Journal of Biostatistics* 6.

Chernozhukov V, Chetverikov D, Demirer M, Duflo E, Hansen C and Newey W (2017) Double/debiased/neyman machine learning of treatment effects. *American Economic Review* 107: 261–65.

Chernozhukov V, Chetverikov D, Demirer M, Duflo E, Hansen C, Newey W and Robins J (2018) Double/debiased machine learning for treatment and structural parameters.

Díaz I, Williams N, Hoffman KL and Schenck EJ (2020) Non-parametric causal effects based on longitudinal modified treatment policies. *arXiv preprint arXiv:2006.01366* .

Haneuse S and Rotnitzky A (2013) Estimation of the effect of interventions that modify the received treatment. *Statistics in Medicine* 32: 5260–5277.

Muñoz ID and Van Der Laan M (2012) Population intervention causal effects based on stochastic interventions. *Biometrics* 68: 541–549.

Newey WK, Hsieh F and Robins JM (2004) Twicing kernels and a small bias property of semiparametric estimators. *Econometrica* 72: 947–962.

Picciotto S, Hernán MA, Page JH, Young JG and Robins JM (2012) Structural nested cumulative failure time models to estimate the effects of interventions. *Journal of the American Statistical Association* 107: 886–900.

Richardson TS and Robins JM (2013) Single world intervention graphs (swigs): A unification of the counterfactual and graphical approaches to causality. *Center for the Statistics and the Social Sciences, University of Washington Series. Working Paper* 128: 2013.

Robins JM (1986) A new approach to causal inference in mortality studies with a sustained exposure period: application to control of the healthy worker survivor effect. *Mathematical Modelling* 7: 1393–1512.

Robins JM, Hernán MA and Siebert U (2004) Effects of multiple interventions. *Comparative quantification of health risks: global and regional burden of disease attributable to selected major risk factors* 1: 2191–2230.

Taubman SL, Robins JM, Mittleman MA and Hernán MA (2009) Intervening on risk factors for coronary heart disease: an application of the parametric g-formula. *International Journal of Epidemiology* 38: 1599–1611.

Van Der Vaart AW (2000) *Asymptotic statistics*, volume 3. Cambridge university press.

Young JG, Cain LE, Robins JM, O'Reilly EJ and Hernán MA (2011) Comparative effectiveness of dynamic treatment regimes: an application of the parametric g-formula. *Statistics in Biosciences* 3: 119–143.

Young JG, Hernán MA and Robins JM (2014) Identification, estimation and approximation of risk under interventions that depend on the natural value of treatment using observational data. *Epidemiologic Methods* 3: 1–19.

Young JG, Logan R, Robins JM and Hernán MA (2019) Inverse probability weighted estimation of risk under representative interventions in observational studies. *Journal of the American Statistical Association* 114: 938–947.
